# *Micromeryx*? *eiselei*—A new moschid species from Steinheim am Albuch, Germany, and the first comprehensive description of moschid cranial material from the Miocene of Central Europe

**DOI:** 10.1371/journal.pone.0185679

**Published:** 2017-10-16

**Authors:** Manuela Aiglstorfer, Loïc Costeur, Bastien Mennecart, Elmar P. J. Heizmann

**Affiliations:** 1 Staatliches Museum für Naturkunde Stuttgart, Rosenstein 1, Stuttgart, Germany; 2 Naturhistorisches Museum Basel, Basel, Switzerland; 3 Naturhistorisches Museum Wien, Vienna, Austria; Institute of Botany, CHINA

## Abstract

Moschids are enigmatic pecoran ruminants whose phylogeny is still not fully understood. So far we know only little of the family’s early evolutionary history and the origin of the modern genus, *Moschus*. Here we present a comprehensive description of cranial material, including the ear region and the dentition, of fossil moschid material from the Middle Miocene locality Steinheim am Albuch (13.5 Ma; Germany). This study provides the first exhaustive dataset for the cranial osteology of *Micromeryx flourensianus*, the most likely oldest true moschid. It furthermore reveals the presence of a second, so far undescribed moschid species, we here name *Micromeryx*? *eiselei*, in the abundant material from the locality. The two taxa can be clearly distinguished by characters of the skull, the ear region, the dentition, as well as by size. This evidences the sympatric occurrence of two moschid species in the locality Steinheim am Albuch.

## Introduction

One of the still intriguing questions in the evolutionary history of ruminants is the phylogeny of the family Moschidae and especially the origin of the modern genus *Moschus* Linnaeus, 1758. Moschidae are small pecoran ruminants with elongated upper canines in males lacking any cranial appendages. *Moschus* remains the only surviving genus of the family. It is restricted to montane woodlands in Asia [1], has been in decline for some decades [2], and was included in the IUCN Red List of Threatened Species [3]. The phylogenetic position of the family has been the subject of debate for a long time: based on morphologic, molecular, and behavioural data, the family was placed at the base of the Pecora [4], or considered as the sister group of either Cervidae (+ Antilocapridae) [5–10], or Bovidae (+Antilocapridae) [11–16] (see also discussions in [9,13,17,18] for this controversy). Although a sister group relationship with bovids appears most likely [11,12,14], the phylogeny of Moschidae is not fully understood with arguments still pointing to a closer relationship with Cervidae (see e.g. discussion in [17]). With only one modern genus, a focus on recent representatives provides a very limited data set highly susceptible to homoplastic features. A better knowledge of the detailed morphology of extinct taxa can improve our understanding of the early evolution of the clade and thus help to better constrain its phylogeny, as also proposed by other authors (see e.g. discussion in [17]). For Miocene Moschidae a comprehensive analysis was undertaken on Spanish material, which showed a quite high diversity with at least five different species in two genera, and a rather common co-occurrence of two sympatric moschid species in one locality [15,19,20]. However, the possibly oldest moschid species, and the type species of the genus *Micromeryx*, *Micromeryx flourensianus* Lartet 1851, remains undescribed so far.

Traditionally dental material was the main source of information for smaller fossil mammals. However, with improved excavation and analysis techniques more data can also be gained on the skeletal morphology of this group. In particular, the area of the basicranium, including the ear region, is often considered highly relevant to resolve phylogenetic relationship, as it is assumed to be less susceptible to climatic and ecological changes [21]. Prior to non-invasive techniques, such as computed-tomography, studies of the ear region that did not partially destroy the skull were limited, and examinations including data of the inner ear of fossil specimens not possible at all. Consequently, few comparative works considered these data sets. However, during the last couple of years, studies on the petrosal and bony labyrinth have proven highly valuable for phylogenetic, developmental, and (palaeo-)ecologic questions in mammals (see e.g. [22–25]), and recently the great value of the ear region was demonstrated also for phylogenetic studies on ruminants [26,27]. A combination of data from this region with observation on other skull elements and the dental morphology can greatly improve our view on fossil taxa and help to better decipher ecomorphologic traits and phylogenetic signals. This might also prove helpful for a better understanding of the early evolutionary history of the Moschidae and the origin of the modern genus *Moschus*.

So far cranial elements of *Micromeryx* have only been described for Spanish species, including the first data on the ear region, but, as mentioned above, data on the type species *M*. *flourensianus* are still missing. Unfortunately the type locality Sansan (about 15 Ma [28]; France) did not deliver any cranial material, including the ear region. However, the Miocene locality Steinheim am Albuch (a. A.; about 13.5 Ma [29]; Germany) yielded very rich material including fairly complete skulls and partial skeletons of moschids, so far considered to represent *M*. *flourensianus*. In a preliminary study focusing on the fossil moschids from the locality Steinheim a. A. Costeur [30] described the petrosal and inner ear of a fossil moschid for the first time and detected considerable variation in the inner ear morphology. As data sets for a reasonable evaluation of intraspecific variation and the value of these data for phylogenetic studies were still limited Costeur [30] proposed to increase taxonomic, ontogenetic, and intraspecific sampling in order to identify evolutionary patterns as a tool to establish phylogenetic relationships. Furthermore, some sparse remains indicated the presence of another, so far unknown, moschid species at the locality [31]. The investigation on the moschid material from Steinheim a. A. presented here represents the first comprehensive investigation on cranial material of Miocene Moschidae from Central Europe, including the analysis of petrosal and inner ear morphology in combination with the study of cranial, mandibular and dental morphology. It reveals that the differences observed by Costeur [30] indeed prove to be of taxonomic value and that two sympatric moschid taxa were present in Central Europe during the Middle Miocene.

### Geographic and geological setting

**Fig 1:**
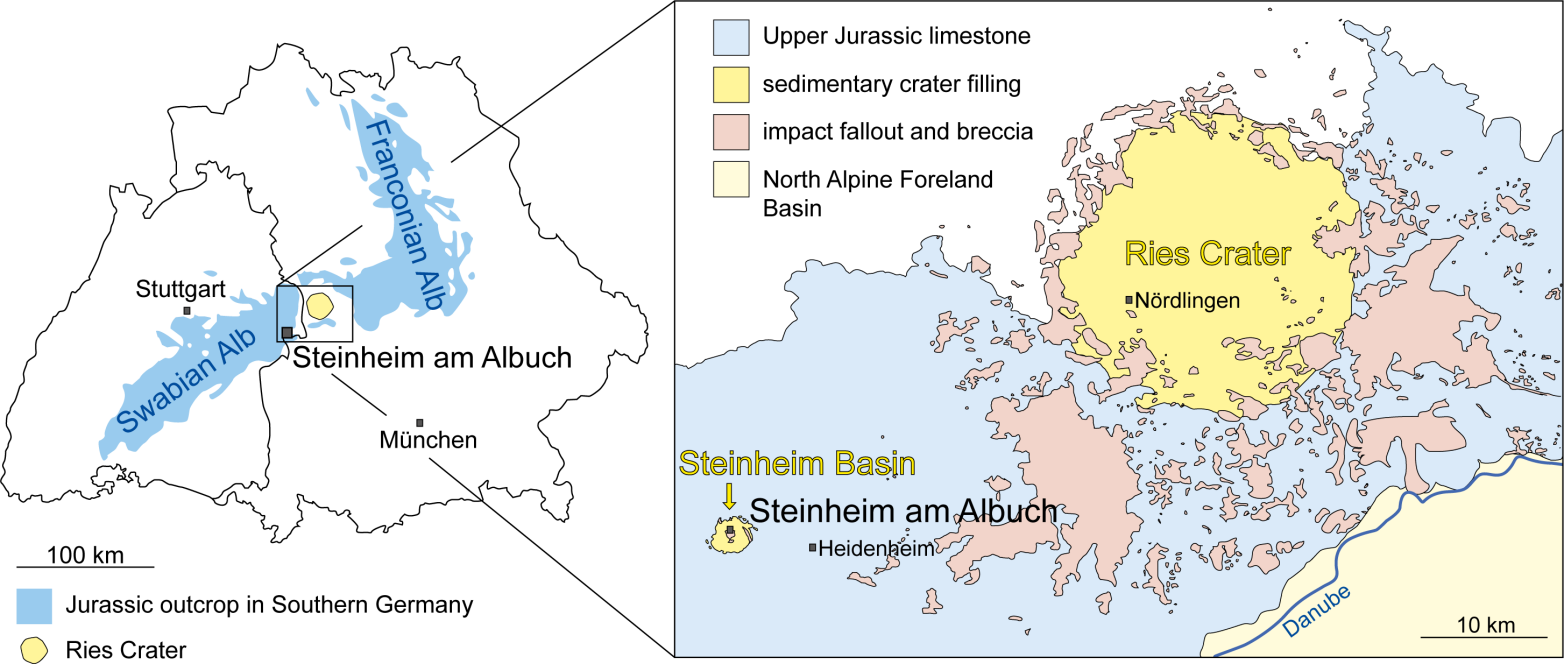
Geographic and geologic setting of the locality Steinheim am Albuch. Outcrop of Jurassic sediments in overview of Germany modified for illustrative purposes after [36]; simplified geologic situation in the area with focus on Steinheim Basin and Ries Crater for illustrative purposes, modified after [37].

The locality Steinheim a. A. is located on the Upper Jurassic Limestone Plateau of the Swabian Alb in Baden-Württemberg, Germany (Fig 1). It comprises Middle Miocene lake sediment in-fills of a crater that formed during a binary asteroid impact 14.6–15.0 Ma ago [32,33].

**Fig 2:**
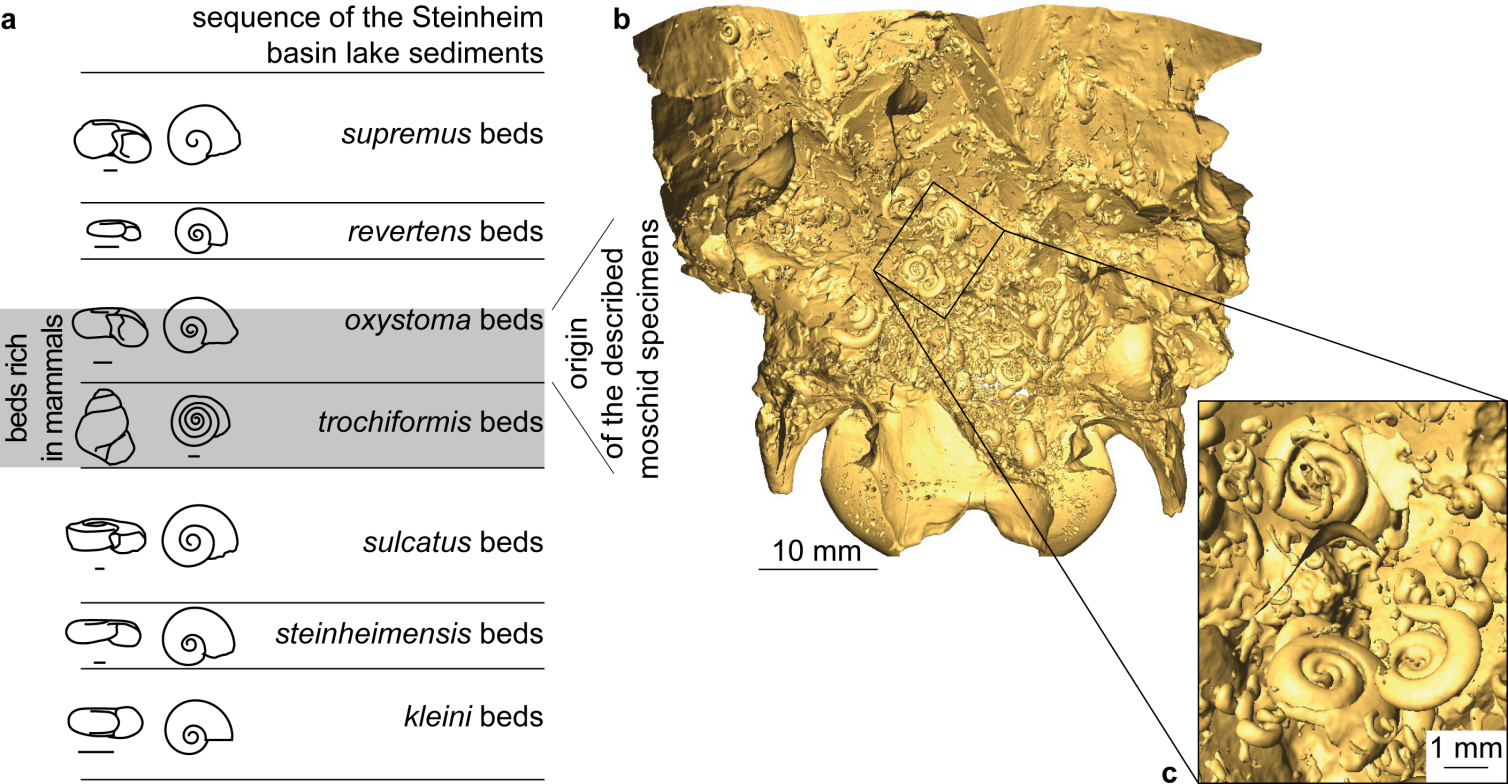
Stratigraphic context of the moschid findings from Steinheim am Albuch. (a) Stratigraphy of the Steinheim Basin lake sediments with sketches of the respective gastropods (drawings after [34]); (b) posterior part of CT-scan of NMB Sth.833, *M*.? *eiselei*, with removed ventral surface showing embedded gastropod shells; (c) detail of sedimentary filling of NMB Sth.833, *M*.? *eiselei*, showing the accumulation of *Gyraulus oxystoma*.

The 30–40 m sedimentary section can be biostratigraphically subdivided in 7 subsections based on the evolutionary development of the gastropod *Gyraulus* (bottom to top: *kleini*-, *steinheimensis*,-, *sulcatus*-, *trochiformis*-, *oxystoma*-, *revertens*-, *supremus*-beds; see Fig 2 and [34] for details). Of these the upper *trochiformis*- to lower *oxystoma*-beds, roughly datable to the Middle Miocene (Serravallian; about 13.5 Ma [29]), are rich in mammal remains (114 vertebrate species described so far [31]).

The first palaeontological investigations on vertebrates from the locality Steinheim a. A. date back to the first half of the 19^th^ century [35]. They mainly focused on larger mammal remains found during commercial quarrying for sand in the community sandpit (former Pharion sandpit) on the west side of the central uplift of the crater. Scientific excavations carried out by one of us (EH) between 1969 and 1982 provided abundant material housed at the Naturhistorisches Museum Basel and the Staatliches Museum für Naturkunde Stuttgart. The most common large mammals found during these excavations are moschids, including several partial skeletons. The investigations presented here are mainly based on this material. Based on the specimens’ sediment infilling, in addition to the field documentation, we can confirm that the material originated from the basal *oxystoma*-beds: an accumulation of the gastropod *Gyraulus oxystoma* is still preserved in the skull of NMB Sth.833 (Fig 2; pers. comm. M. Rasser (17.03.2017)) and it is also present in NMB Sth.834 and in NMB Sth.811.

## Material and methods

### Material

The moschid material described in this work, including the new species *Micromeryx*? *eiselei*, comes from the Middle Miocene locality Steinheim am Albuch (Germany) and is housed in the Staatliches Museum für Naturkunde Stuttgart and the Naturhistorisches Museum Basel (the specimens referred to in this publication do not comprise the entire moschid material from Steinheim am Albuch, but are a selection with focus on cranial material; postcranial material exists and will be the focus of another study; for referred material see respective sections in the chapter Systematic Palaeontology). All necessary permits were obtained for the described study, which complied with all relevant regulations.

In order to understand the morphological traits in the moschid taxa from Steinheim a. A., different ruminant taxa were included in the comparison and the phylogenetic analysis.

For Moschidae the following recent *Moschus*-specimens were used for comparison: SMNS 143 (*Moschus moschiferus*, male, Siberia), SMNS 1238 (*M*. *moschiferus*?, male, Siberia?), NMB 5111 (*M*. *moschiferus*, male, juvenile, Altai), NMB 5112 (*M*. *moschiferus*, female, Altai), NMB 5731 (*M*. *moschiferus*, male, juvenile, Altai), NMB 5732 (*M*. *moschiferus*, male, Altai), NMB 8874 (*M*. *moschiferus parvipes*, male, Korea), NMB 5110 (*M*. *moschiferus*, male, Altai), and NMB C 4201 (*M*. *moschiferus*, male). Furthermore, literature data [15,19] was considered for *Micromeryx azanzae* Sánchez and Morales, 2008 and *Hispanomeryx daamsi* Sánchez, Domingo and Morales, 2010.

The earliest representatives of fossil artiodactyls *Diacodexis ilicis* Gingerich, 1989 (coding after descriptions and figures in [27,38–40] and *Diacodexis* ssp. in [41]) and *Homacodon vagans* Marsh, 1872 (coding after descriptions and figures in [27,40,42]) were chosen as outgroups. For comparison with ruminants we chose the non-pecoran tragulids *Dorcatherium crassum* (Lartet, 1851) (fossil; data from [43,44], as well as observation of material housed at SMNS and MNHN), and *Tragulus javanicus* (recent; data from personal observation on SMNS 38889; NMB 10028), the cervids *Procervulus dichotomus* (Gervais, 1849) (fossil; data from [45] and personal observation on SMNS 41063 (*Procervulus* sp.)), *Heteroprox larteti* (Filhol, 1890) (fossil; data from personal observation on SMNS 9967, 43320, 47709, 42853, 47708, and 42853, MNHN Sa 3317 and 3399), *Dicrocerus elegans* (Lartet, 1837) (fossil; data from personal observation of dental material at SMNS and MNHN as well as of MNHN 840(?) and 3379), *Euprox furcatus* (Hensel, 1859) (fossil; data from [46] and personal observation on material housed at SMNS, GPIT and UMJGP), and *Cervus elaphus* (recent; personal observation on SMNS 16904, SMNS 16908, SMNS 46614, SMNS 38899). For the morphology of the petrosal and the inner ear of these taxa we refer to the data from [27].

### Methods

Data for the ear region were acquired by scanning NMB Sth.828a, NMB Sth.865, NMB Sth.866, SMNS 42920, SMNS 43062, NMB Sth.833, NMB Sth.834, and SMNS 40010 with high resolution x-ray computed tomography at the Biomaterial Science Centre of the University of Basel using a phoenix nanotom® (General Electric Wunstorf, Germany) equipped with a 180 kV / 15 W nanofocus x-ray source. Various scanning resolutions were employed based on specimen sizes, densities and scan measurement time: NMB Sth.833 was scanned at a 60 μm resolution; NMB Sth.834, SMNS 42920, and SMNS 40010 at 36 μm; SMNS 43062 at 45 μm; NMB Sth.828a at 30 μm; NMB Sth.865 and NMB Sth.866 at 18.5 μm. Raw data are available upon request.

For SMNS 40500–1, SMNS 40252, SMNS 46123, data for the ear region were acquired by scanning with x-ray computed tomography at the Staatliches Museum für Naturkunde Stuttgart using a Bruker Skyscan 1272 equipped with a 20-100kV / 10W x-ray source.

3D reconstructions of inner ears and petrosals were achieved using the segmentation editor of software AVIZO® 7.0 and AMIRA® 6.2. Difference in resolution only has minor impact on the reconstructions of petrosals or on the segmentation of inner ears. Three-dimensional data are provided by [47].

Measurements of teeth were done with digital callipers. Precision of measurements is 0.2 mm.

See supporting information for dental measurements.

The phylogenetic analysis presented here is based on a matrix of 50 characters, comprising 10 characters from the petrosal bone, 11 characters from the bony labyrinth, 19 characters from the cranium and mandibula, and 10 dental characters. We combined characters traditionally used to structure the basal nodes of the phylogenetic tree of ruminants with characters diagnostic for the moschid material from Steinheim a. A. The main purpose was to test the phylogenetic relationships of the two taxa from Steinheim a. A. with the extant moschid *Moschus moschiferus*. The analysis was performed using WinClada [48]. All characters were equally weighted without any ordering. All multistate characters were treated as unordered. We ran an exhaustive search which resulted in only one tree of 35 steps. See supporting information for the character list and character matrix used in the phylogenetic analysis.

Terminology for the cranium and mandibula follows [49] and [50], for petrosal and inner ear [24], [30], and [27]. For the dentition we refer to [51]. Due to the more common use in recent literature, anterior and posterior are used instead of mesial-distal and rostral-nuchal.

Nomenclatural acts

The electronic edition of this article conforms to the requirements of the amended International Code of Zoological Nomenclature, and hence the new names contained herein are available under that Code from the electronic edition of this article. This published work and the nomenclatural acts it contains have been registered in ZooBank, the online registration system for the ICZN. The ZooBank LSIDs (Life Science Identifiers) can be resolved and the associated information viewed through any standard web browser by appending the LSID to the prefix "http://zoobank.org/". The LSID for this publication is: urn:lsid:zoobank.org:pub:86EDF102-759B-4A77-83C7-7FAE99074C5A. The electronic edition of this work was published in a journal with an ISSN, and has been archived and is available from the following digital repositories: PubMed Central, LOCKSS.

### Systematic palaeontology

MAMMALIA Linnaeus, 1758

CETARTIODACTYLA Montgelard, Catzeflis and Douzery, 1997

RUMINANTIA Scopoli, 1777

MOSCHIDAE Gray, 1821

Genus *Micromeryx* Lartet, 1851

Type species: *Micromeryx flourensianus* Lartet, 1851

### Micromeryx flourensianus

Holotype: hitherto not determined (Ginsburg not formally proposed (letter from 1974) MNHN Sa 2957); material from Sansan (France, MN6) under revision; partly figured in Filhol (1891, pls. 24, 25); stored at MNHN.

#### Material considered in this analysis

NMB Sth.834 (compressed skull of skeleton); Sth.804 (fragments of upper and lower jaw of skeleton), NMB Sth.811 and Sth.812 (same individual; skull fragment with dentition); NMB Sth.828a, NMB Sth.865, NMB Sth.866, SMNS 40500–1 (all isolated petrosals), SMNS 46123 (lower jaw, canines, and petrosal), SMNS 15776 (fractured palate), SMNS 12966 (fragmented mandibula with worn and fragmented post canine toothrow and incisor fragments), SMNS 46116 (right lower jaw fragment), SMNS 40252 (fragment skull with petrosal and teeth), SMNS 42920 (fragment of posterior part of skull with petrosal and some isolated teeth), SMNS 43062 (skull fragments with petrosal), SMNS 46083 (maxilla fragment).

NMB Sth.834 and NMB Sth.865, NMB Sth.866, SMNS 40500–1, SMNS 46123 comprise adult specimens. NMB Sth.828a and NMB Sth.811 were juveniles or at young adults. The more complete skull NMB Sth.834 was enclosed obliquely and was strongly fractured and fragmented.

#### Description and comparison

Generally *Micromeryx flourensianus* from Steinheim a. A. possessed an elongated and low skull, with a broadened cranium and a facial part distinctly narrowing to anterior, similar to the modern genus *Moschus*. As the best preserved skull (NMB Sth.834) is strongly compressed estimations on the skull size are enhanced, but it appears to have been only slightly smaller than the modern *Moschus moschiferus*. A roughly rounded shape can be reconstructed for the orbita (observable in NMB Sth.834 and NMB Sth.811). Its relative size (in ratio to skull size) appears a little larger than in the modern *Moschus*.

**Fig 3:**
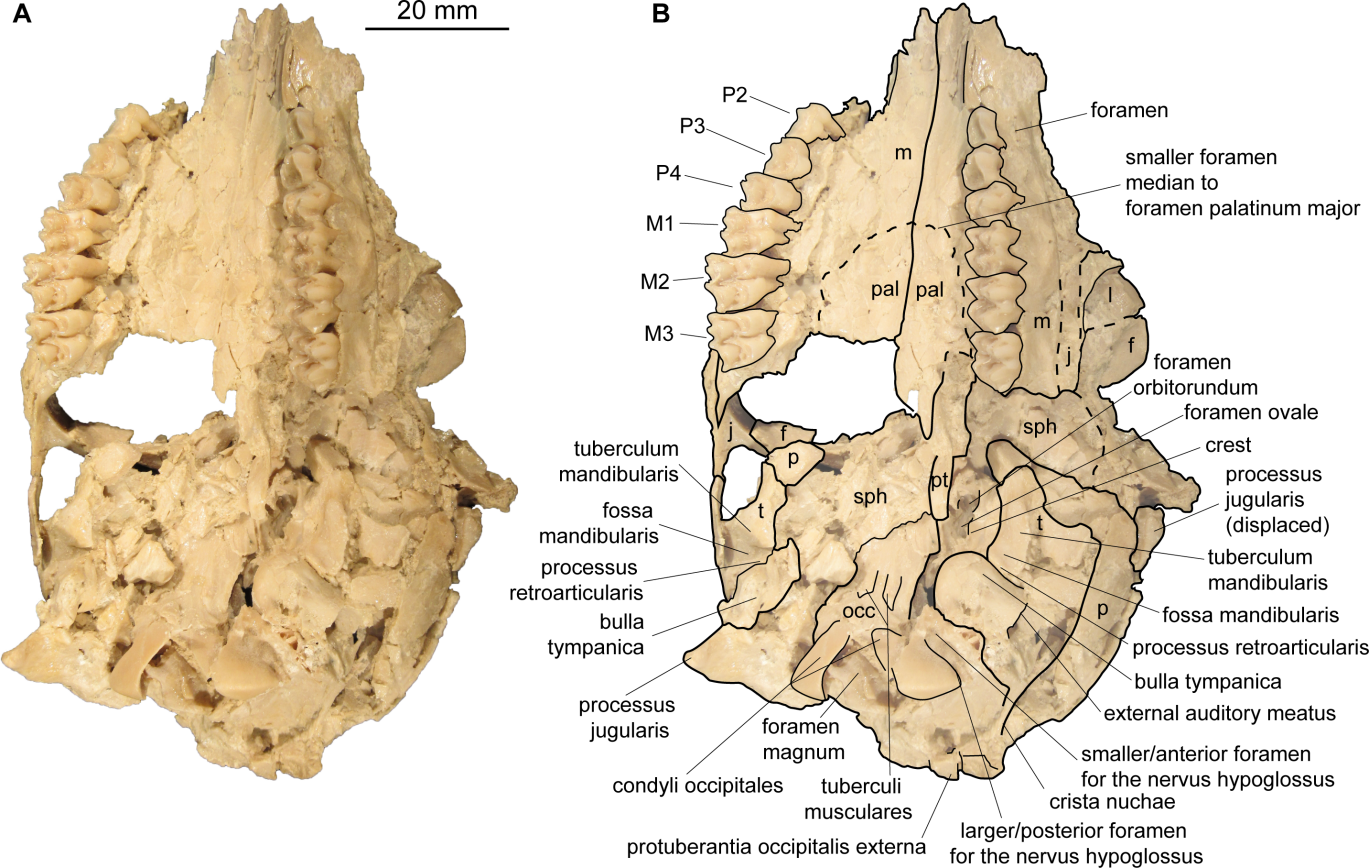
Skull of *Micromeryx flourensianus* from Steinheim a. **A. (**a) Ventral view of NMB Sth. 834; (b) same as a with labelling of anatomic features; see material and methods section for abbreviations.

**Fig 4:**
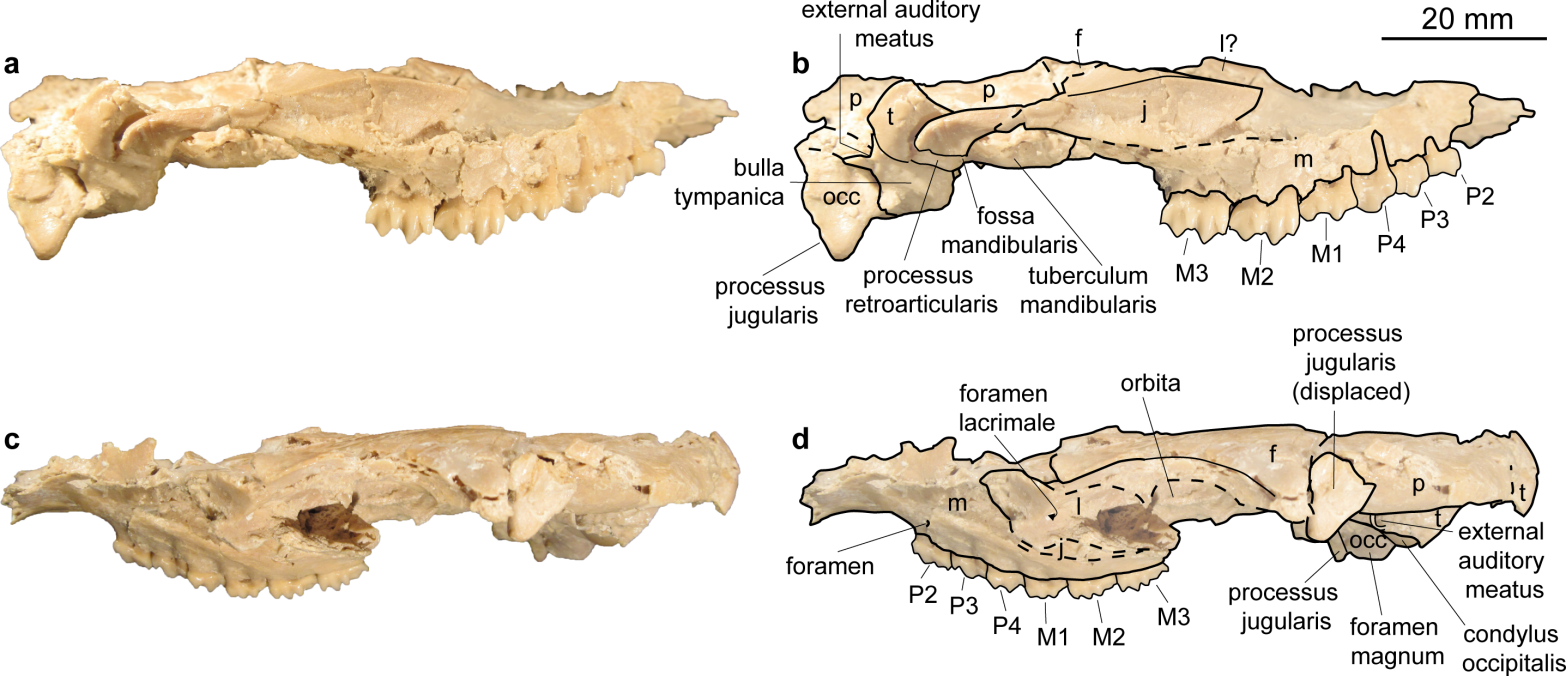
Skull of *Micromeryx flourensianus* from Steinheim a. **A.** (a) dextral view of NMB Sth. 834; (b) same as a with labelling of anatomic features; (c) sinistral view of NMB Sth. 834; (d) same as c with labelling of anatomic features; see material and methods section for abbreviations.

Occipital (Figs 3 and 4). The partes laterales and the squama occipitalis of the occipital can be observed in NMB Sth.834, where they are obliquely compressed. A narrow, but distinct crista nuchae (as in *Micromeryx azanzae* [19]) appears to meet a well pronounced protuberantia occipitalis externa, comparable to the condition in *Moschus* and *Hispanomeryx daamsi* [15]. The area around the condyli occipitales and the shape of the foramen magnum are best preserved in SMNS 42920; however, due to compression the actual shape of the foramen cannot be reconstructed. A clear tuberculum nuchale is present on both sides of the dorsal rim lateral of a median incision. The processus jugularis is triangular and short as in *Moschus* but slightly more robust than in the latter. It is not as long and slender as e.g. in *C*. *elaphus*. Due to compression the size and position of the foramen jugulare cannot be reconstructed. There are two foramina for the nervus hypoglossus. The larger and posterior foramen is positioned more posterior in the fossa condylaris ventralis than it is generally the case in *Moschus* and *C*. *elaphus*. There is no foramen posterior to it in medial view as it is often the case in *Moschus* and *C*. *elaphus*.

The pars basilaris shows a smooth surface, except of the two concave surfaces for the muscle insertion with the distinct posteromedial tuberculi musculares and the posterior protuberantia at the suture to the partes laterales (Fig 3). We could not reconstruct the suture line with the basisphenoid. The tuberculi musculares are more posterior than they are usually in *C*. *elaphus*.

**Fig 5:**
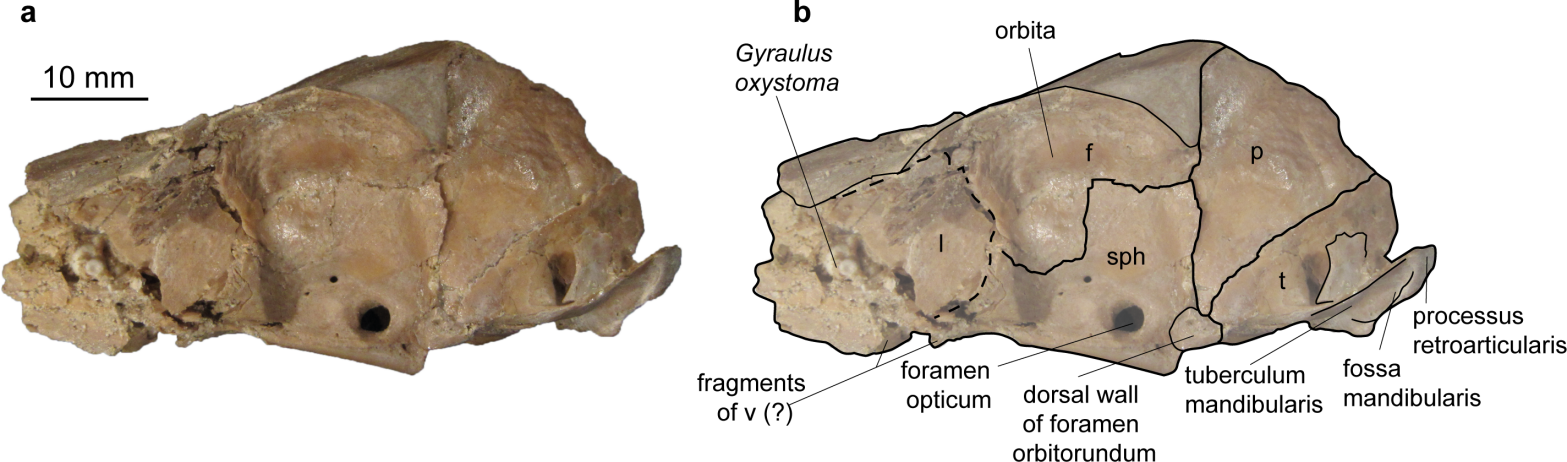
Skull of *Micromeryx flourensianus* from Steinheim a. **A.** (a) sinistral view of NMB Sth. 811; (b) same as a with labelling of anatomic features; see material and methods section for abbreviations.

Sphenoid (Figs 3 and 5). In NMB Sth.811 the ala ossis presphenoidalis forms the lower part of the medial wall of the orbita and meets the frontal here in an s-shaped suture line with the frontal reaching more ventral anteriorly. As in *Moschus* the ala ossis presphenoidalis is dorsoventrally higher than in *C*. *elaphus*, where the palatine more strongly intrudes into the medial wall of the orbita in dorsal direction. A relatively large foramen opticum (of similar size as in *Moschus*; relative to the skull size larger than in *C*. *elaphus*) is located posterior to two smaller foramina. The anteroposteriorly oriented foramen orbitorundum is observable in NMB Sth.811 (Fig 5). Posterolaterally the sphenoid meets the parietal and the temporal. The crest formed by sphenoid, vomer and pterygoid is preserved in ventral view. In NMB Sth.834 this area is heavily crushed, but the rests of the foramen orbitorundum and foramen ovale are still discernible median to a small crest (Fig 3). The latter could represent the weak crest described as diagnostic for Moschidae by Sánchez et al. [15] inside Bovoidea.

Temporal. Pars squamosa (Figs 3 and 4). The processus zygomaticus with fragments of the tuberculum articulare, the fossa mandibularis and the processus retroarticularis is preserved on the pars squamosa in NMB Sth.834. The processus retroarticularis comprises a well pronounced mediolateral crest, as in *Moschus*. The long and slender processus zygomaticus reaches as anterior as in *Moschus*. The suture with the jugal cannot be defined clearly as the respective part is heavily broken and covered with glue. The suture line to the parietal can be roughly followed in ventral view of NMB Sth.834, where the lateral part is partly shown due to the oblique compression (Fig 3), and in the lateral view of NMB Sth.811 (Fig 5). The suture line ascends from anterolaterally to posteromedially. It is more ventral than in *C*. *elaphus*, but similar to the condition in *Moschus*.

**Fig 6:**
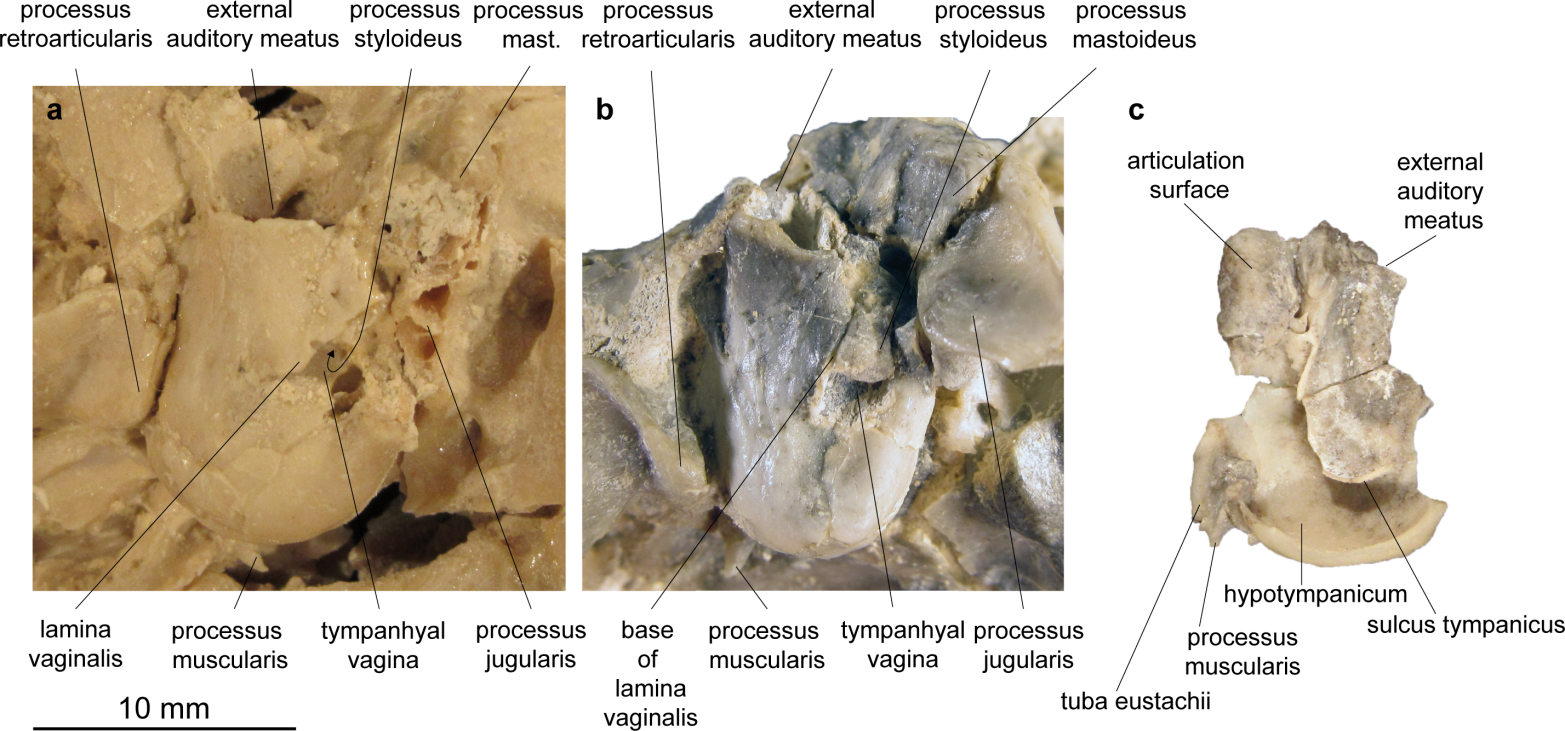
Bullae of Moschidae from Steinheim a. **A.** (a) left bulla of NMB Sth.834, *M*. *flourensianus* from Steinheim a. A., in ventrolateral view; (b) left bulla of NMB Sth.833, *M*.? *eiselei*, in ventrolateral view; (c) right bulla of NMB Sth.833, *M*.? *eiselei* in medial view.

Pars mastoidea and tympanica (Fig 6). The processus mastoideus is well developed and the temporal forms at least the ventral half of the crista nuchae as in *Moschus* and *Hispanomeryx daamsi* [15]. The bulla tympanica in NMB Sth. 834 is more strongly inflated than in *Moschus* and slightly more than in *M*.? *eiselei*. Due to compression it cannot be verified if the bulla tympanica covered the foramen retroarticulare. The ventral surface is rounded. The external auditory meatus is large and circular. The bulla is not strongly posteriorly expanded, the tympanohyal vagina appears slightly more posterior than in *M*.? *eiselei* (that this is influenced by different compression cannot be ruled out however). The lamina vaginalis encloses the processus styloideus at least proximally. Thus the thympanohyal vagina is not fully visible from lateral view. However, as the lamina is fragmented the full extent cannot be reconstructed. The processus styloideus is separated from the processus jugularis of the occipital, comparable to the situation in *Moschus*. In *C*. *elaphus* this feature was variable in the studied specimens. There appears to be no contact between the tympanic bulla and the occipital in *M*. *flourensianus* from Steinheim a. A.

**Fig 7:**
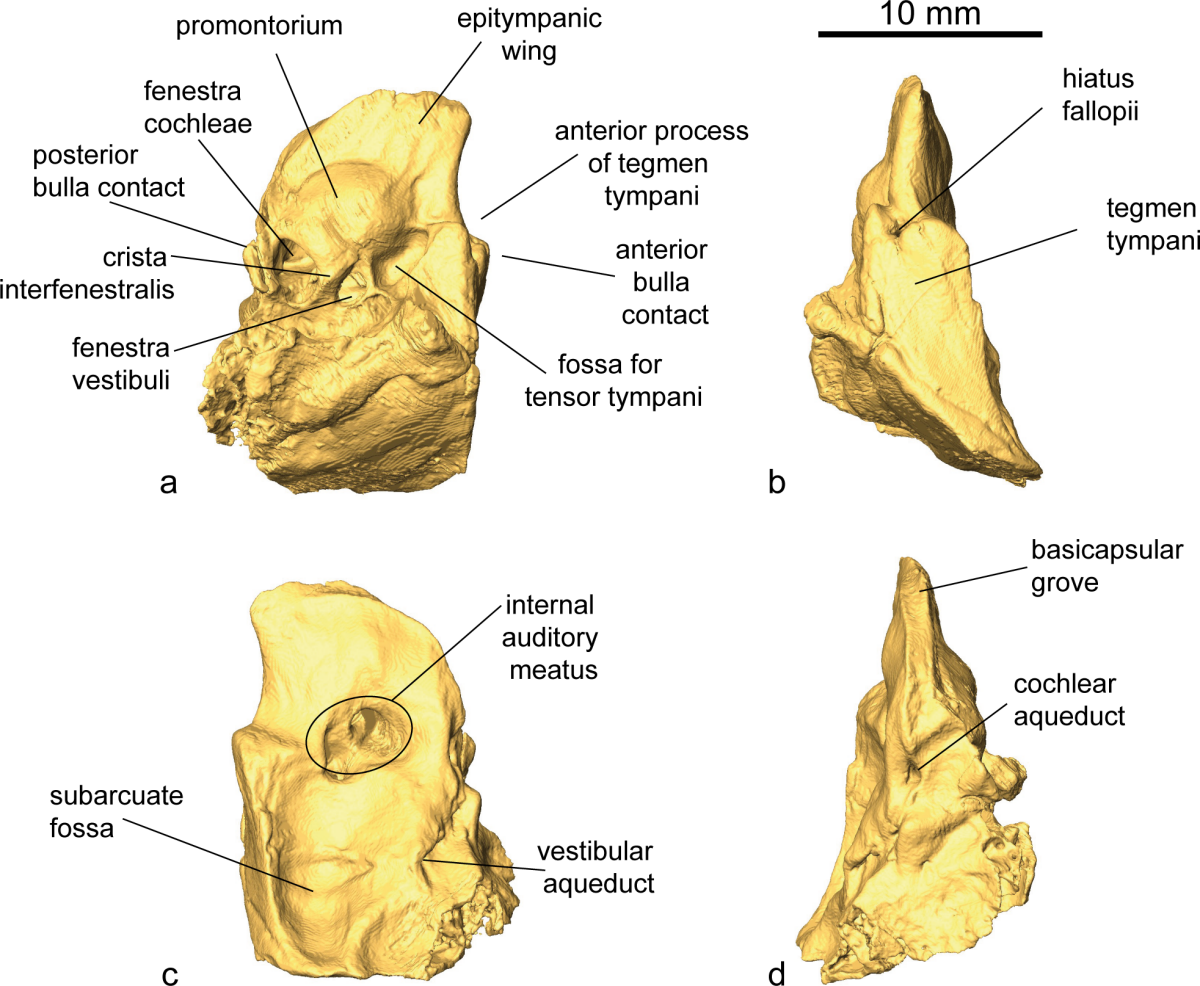
Petrosal of *M*. *flourensianus* from Steinheim a. **A.** 3D reconstruction of left petrosal of NMB Sth.834 (a) ventrolateral view; (b) dorsolateral view; (c) dorsomedial view; (d) ventromedial view.

**Fig 8:**
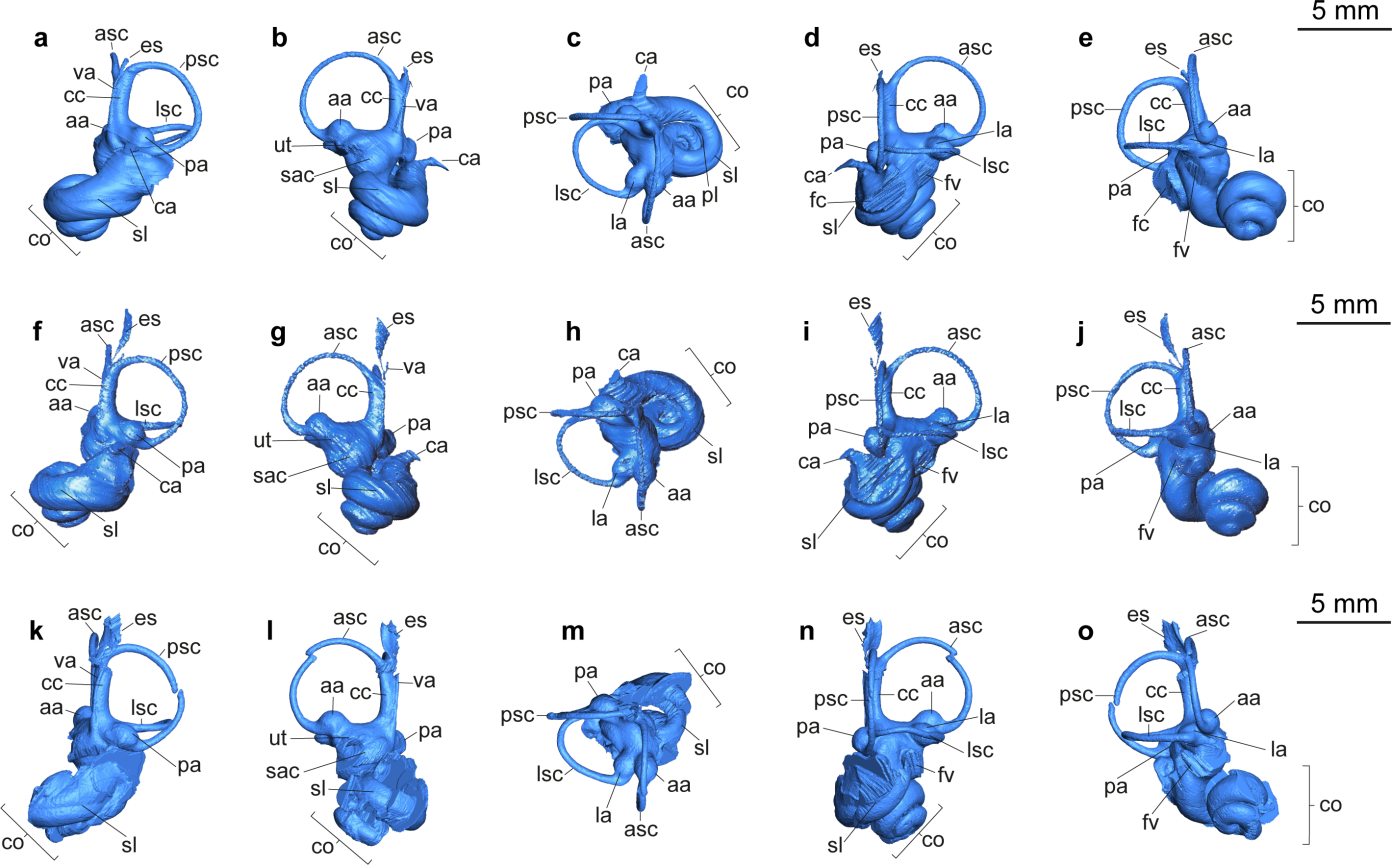
3D reconstruction of bony labyrinths of Moschidae from Steinheim a. **A.** (a-e) NMB Sth.834, *M*. *flourensianus* from Steinheim a. A. (mirrored); (f-j) NMB Sth.833, *M*.? *eiselei* (modified after [30]); (k-o) SMNS 40010, *M*.? *eiselei* (mirrored); (a, f, k) occipital view; (b, g, l) medial view; (c, h, m) dorsal view; (d,i,n) lateral view; (e,j,o) rostral view; see material and methods for abbreviations.

Pars petrosa and bony labyrinth (Figs 7 and 8). The petrosal and bony labyrinth of *M*. *flourensianus* from Steinheim a. A. were both described in detail by [30]. The specimen NMB Sth.834 was not known back then but its morphology is highly similar to NMB Sth.828a, NMB Sth.865 and NMB Sth.866 with a more ventrally facing basicapsular groove and a bean shaped fossa for tensor tympani. The transpromontorial sulcus is not obvious on the promontorium of NMB Sth.834 but a slight groove towards the epitympanic wing may show it was present. The anterior process of the tegmen tympani is relatively shorter and blunter than in NMB Sth.828a, NMB Sth.865 and NMB Sth.866 and the tegmen tympani itself is slightly more expanded. The dorsomedial aspect is virtually identical. The bony labyrinth is also virtually identical with the ones published before with a relatively shorter vestibular aqueduct and endolymphatic sac than in *M*.? *eiselei* (see description below). The aqueduct is slightly curved along the common crus both on NMB Sth.834 and SMNS 46123. The cochlea has 2.25 to 2.5 turns, in the range of what is known for *M*. *flourensianus* from Steinheim a. A. [30]. It has the typical high insertion of the lateral semi-circular canal in the posterior ampulla, the massive cochlea and the short and rounded cochlear aqueduct. The bony labyrinths NMB Sth.834 and SMNS 46123 fully fit within the variability of *M*. *flourensianus* from Steinheim a. A. (for further details on the petrosal and bony labyrinth see [30]).

**Fig 9:**
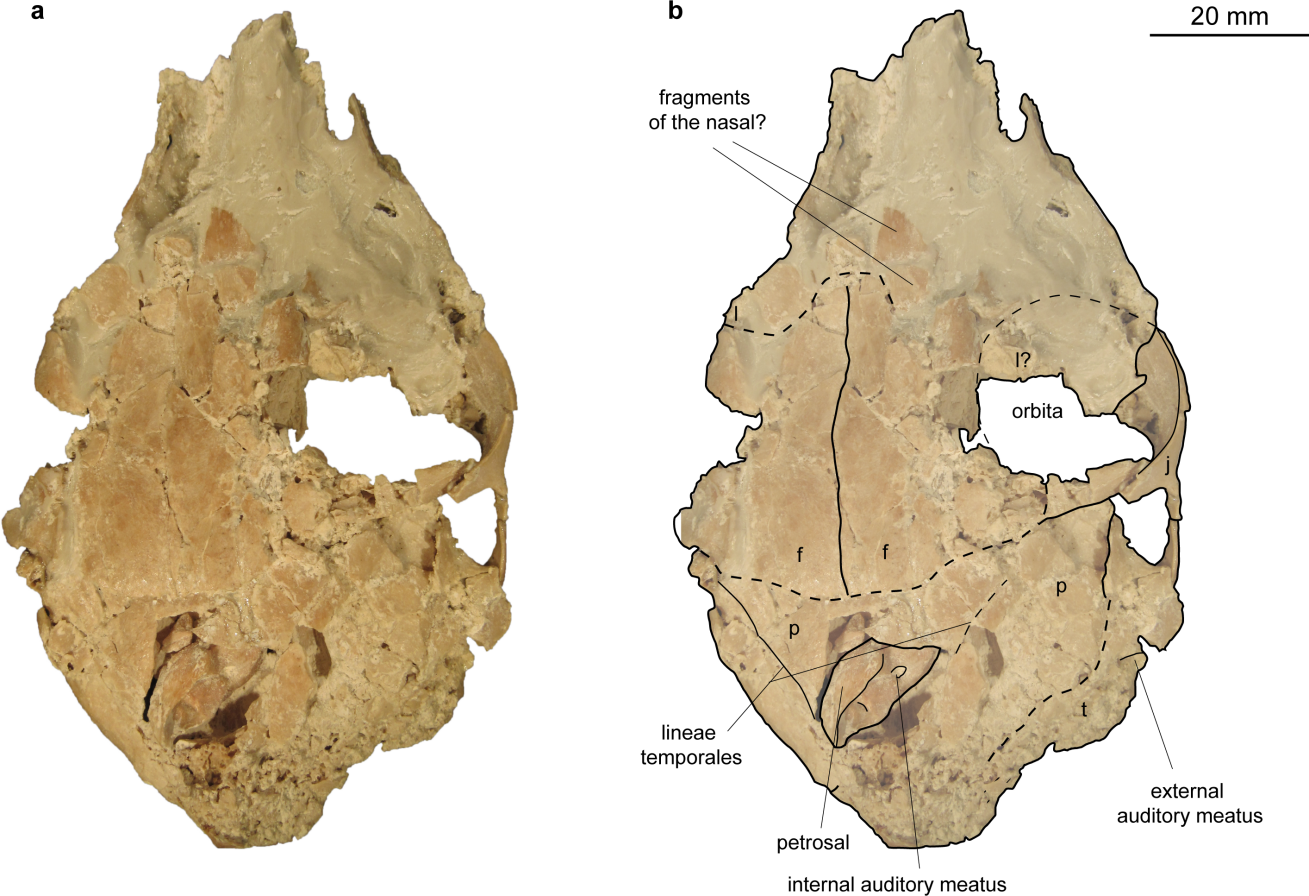
Skull of *Micromeryx flourensianus* from Steinheim a. **A.** (a) dorsal view of NMB Sth. 834; (b) same as a with labelling of anatomic features; see material and methods for abbreviations.

Parietal (Figs 3, 4, 5 and 9). The parietal is preserved fragmentarily in NMB Sth.834 and NMB Sth.811. The linea temporalis and the crista sagittalis externa are weaker than in *M*.? *eiselei*. However, presence, extent and strength of the latter varies in *Moschus* (a clearly distinct crista sagittalis was e.g. only observed in SMNS 1238).

Frontal (Figs 3, 4, 5 and 9). The paired frontals form the interorbital part of the skull roof, as well as the dorsal and most of the medial wall of the orbitae. There are no indications for cranial appendages in any specimen. The supraorbital frontal convexity and the depression anterior to it cannot be reconstructed due to compression. However, based on NMB Sth. 811 they appear similar to the condition in *Moschus*, *Micromeryx azanzae* [19] and *Hispanomeryx daamsi* [15]. The foramen ethmoidale and the part of the skull with the anterior part of the frontal are not preserved in any of the specimens. Presence and extent of the fontanella nasolacrimalis (etmoidal vacuity in [15]; antorbital vacuity in [10]) cannot be reconstructed, therefore.

A well pronounced depression (oriented anteroposteriorly; posteriorly overlapping with the suture line to the sphenoid) is preserved in the orbita of NMB Sth.811. The dorsal rim of the orbita is more filigree than in *M*.? *eiselei*. The foramina supraorbitalia are not as distinct in *M*. *flourensianus* from Steinheim a. A. as they are in *M*.? *eiselei*. A weak sulcus supraorbitalis is preserved in *M*. *flourensianus* from Steinheim a. A. (see NMB Sth.811). The postorbital bar is preserved fragmentarily. As in *Moschus* the frontal seems to form the dorsal 2/3 of the bar. It appears slightly wider mediolaterally than in *Moschus*.

Ethmoid. Fragments of the ethmoid can be observed in NMS Sth.811 and in the CT scan of NMB Sth.834 though specific characteristics of the bone are not discernible.

Nasal (Fig 9). Fragments of the nasal are preserved in NMB Sth.834 but the precise morphology cannot be reconstructed.

Lacrimal (Figs 3, 4, 5 and 9). A fragmented and compressed lacrimal is preserved in NMB Sth.834 and NMB Sth. 811. It forms the anterior rim of the orbita. The facies facialis is slightly concave, but, as in *Moschus* and *Micromeryx azanzae* [19], it does not form a distinct fossa lacrimalis. On the facies orbitalis a small fossa sacci lacrimalis houses one foramen lacrimale just inside the orbit, comparable to the condition in *Moschus*.

Jugal (Figs 3 and 4). Shape and size of the jugal cannot be fully reconstructed due to compression. It forms the ventral rim of the orbit. Like in *Moschus* and *Micromeryx azanzae* [19] the crista facialis is well marked. It is stronger than in *C*. *elaphus*. The participation of the jugal on the postorbital bar cannot be estimated reliably; we nonetheless found no indication that it exceeds more than 1/3 of the total bar in any of the specimens. The processus temporalis is preserved on the right side of NMB Sth.834. It appears to reach not more than the anterior 1/3 of the zygomatic arch and is laterally overlain by the temporal.

**Fig 10:**
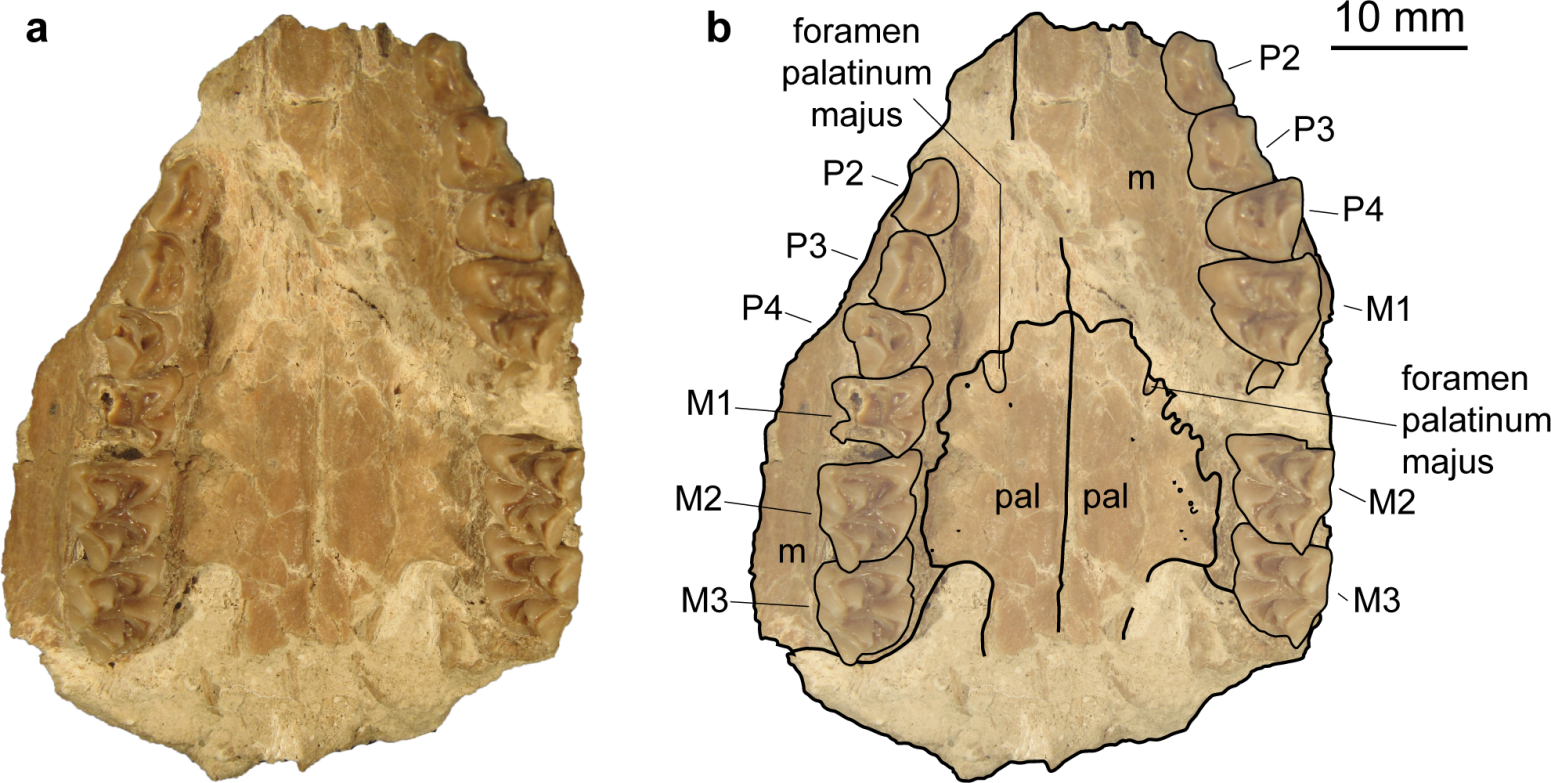
Palate of *Micromeryx flourensianus* from Steinheim a. **A.** (a) ventral view of SMNS 15776; (b) same as a with labelling of anatomic features; see material and methods for abbreviations.

Maxilla (Figs 3, 4, 9 and 10). The anterior part of the maxilla is not preserved for *M*. *flourensianus* from Steinheim a. A. The posterior part of the facies facialis is smooth and slightly convex in NMB Sth.834. It meets the ventral side anterior of the tooth row forming a sharp, delicate crest. Due to compression it cannot be verified, if the crista facialis proceeds onto the maxilla. There appears to be a small foramen at the height of the posterior root of the P2 (observable in NMB Sth.834; Fig 4). It might be the infraorbital foramen. As it is rather small and as the area anterior to the P2 is fractured it cannot be ruled out, however, that a larger infraorbital foramen is located more anterior, as it is the case in *Micromeryx*? *eiselei*. The lacrimal-jugal suture lines are concealed due to compression; they roughly show an anterodorsal to posteroventral course. The facies pterygopalatina is laterally compressed in NMB Sth.834, but was not wider than 6–10 mm anterior of the P2. The maxillae meet along the midline and suture with the palatines at a curved line running from the midline at about the height of the P4 posterolaterally to the height of the M3 (well observable in SMNS 15776 (Fig 10); hardly in NMB Sth.834 (Fig 3)). Thus the palatine protrudes more anteriorly in this taxon than in *Moschus*, where the palatine reaches only as far as the M1.

Praemaxilla. The praemaxilla is not recorded for *M*. *flourensianus* from Steinheim a. A.

Palatine (Figs 3 and 10). Anteriorly bordering the maxilla the lamina horizontalis of the palatine forms the posterior part of the bony palate including the anterior rim of the choanae. As mentioned above the palatine reaches further anterior than in *Moschus*. A fairly large foramen palatinum majus (observable in SMNS 15776 (Fig 10)) is positioned at the anterior suture line with the maxilla, as is the case in *Moschus* (often reaching further anterior on the maxilla in this genus, however). This area is strongly fractured in NMB Sth.834 but a smaller foramen median to the foramen palatinum majus can still be seen. Several small foramina on the palatine are preserved in SMNS 15776. In NMB Sth.834 and SMNS 15776 the posterior rim of the lamina horizontalis is rounded (concave to anterior). Laterally it meets the maxilla at the height of the M3 (see description of maxilla for general course of suture line), medially the lamina perpendicularis penetrates posteriorly to about the height of the foramen opticum, forming the lateral wall of the choanae. As in *Moschus* (observable in NMB Sth.811; Fig 5) it is less high than in *C*. *elaphus*, where it reaches further dorsal than in moschids. Fragments of the fossa pterygopalatina are preserved posteromedially of the tooth row in NMB Sth.834.

Pterygoid. Fragments of the pterygoid are preserved in NMB Sth. 834. No statement can be given on extent and morphology however.

Vomer. In NMB Sth.811 possible fragments of the vomer are still preserved. Statements on shape and posterior extent cannot be given.

**Fig 11:**
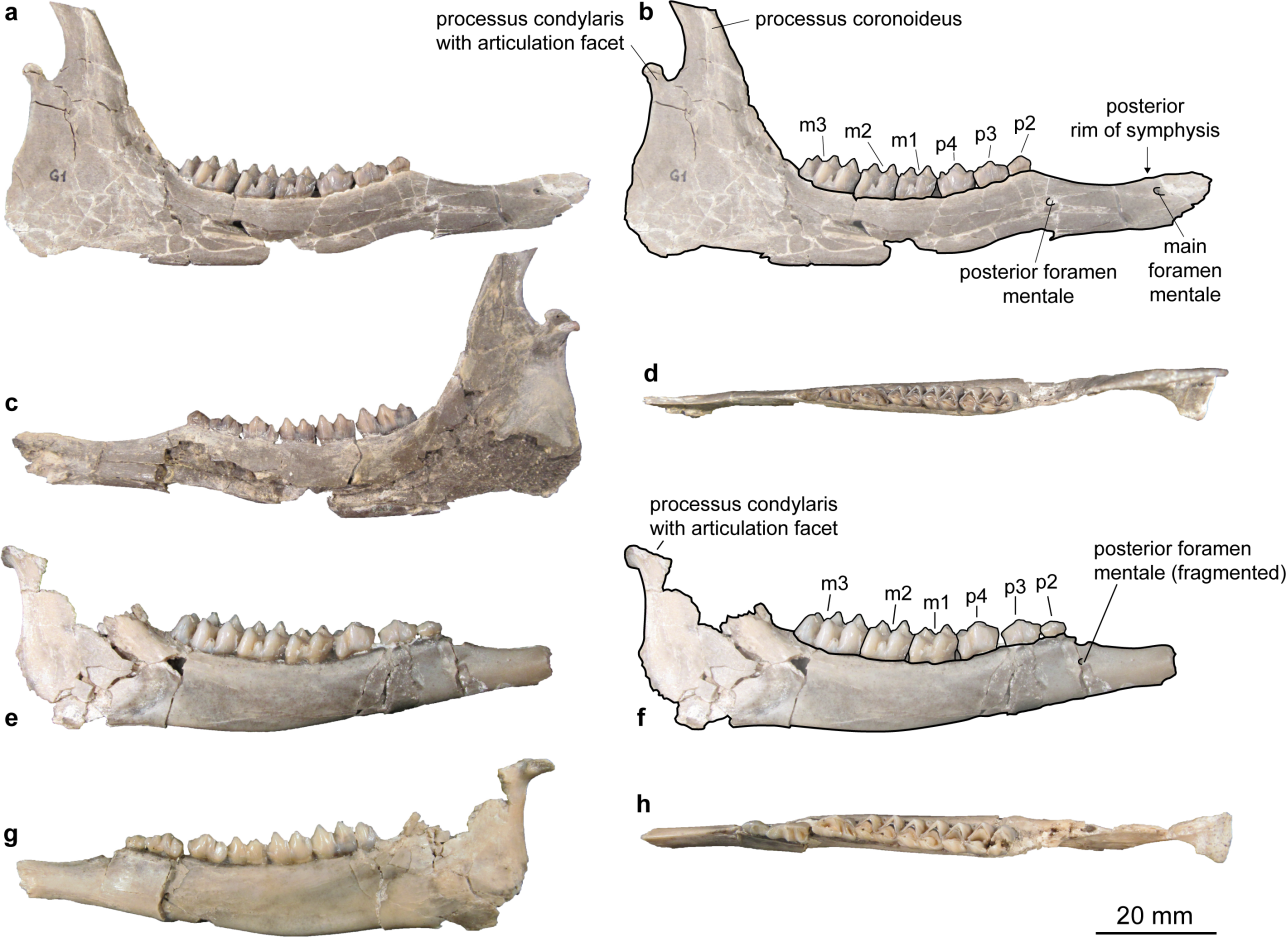
Mandibulae of Moschidae from Steinheim a. **A.** (a) labial view of SMNS 46123, *M*. *flourensianus* from Steinheim a. A.; (b) same as a with labelling of anatomic features; (c) lingual view of SMNS 46123, *M*. *flourensianus* from Steinheim a. A.; (d) occlusal view of SMNS 46123, *M*. *flourensianus* from Steinheim a. A.; (e) labial view of SMNS 40010, *M*.? *eiselei*; (f) same as e with labelling of anatomic features; (g) lingual view of SMNS 40010, *M*.? *eiselei*; (h) occlusal view of SMNS 40010, *M*.? *eiselei*.

Mandibula (Fig 11). *Micromeryx flourensianus* from Steinheim a. A. possesses a slender corpus mandibulae with a long convex ventral side as in *Moschus* and *C*. *elaphus* (see e.g. SMNS 46123). SMNS 12966 shows a fragmented pars incisiva widening to anterior. The symphysis mandibulae is preserved only fragmentarily; its length is about 30 to 40% of the length of the lower post canine tooth row. The relative length of the margo interalveolaris appears shorter than in *Moschus* (line measured from anterior rim of p2 to posterior rim of symphysis about 40 to 50% of the lower post canine tooth row in *M*. *flourensianus* from Steinheim a. A., while it is usually about 50 to 60% in *Moschus*). The main foramen mentale, opening anteriorly, is large and situated at the height of the posterior rim of the symphysis mandibulae or slightly posterior to it on the facies labialis, as in *Moschus*. *M*. *flourensianus* from Steinheim a. A. often possesses a second, smaller foramen mentale situated at the anterior part of the premolar row or just anterior to it (observable in NMB Sth. 804, SMNS 12966, SMNS 46123, and SMNS 46116). In *Moschus*, this smaller foramen is, when present, positioned more anteriorly, at the diastema just posterior to the larger one. The incisura vasorum facialium is fragmented in every specimen. Shape and extent of the angulus mandibulae is comparable to the condition in *Moschus*. The ramus mandibulae is relatively long in anteroposterior direction, as is the processus coronoideus, in comparison to *Moschus*. The processus condylaris possesses a short collum mandibulae and thus the incisura mandibulae is shallow. The articulation facet is often preserved obliquely due to compression. Nevertheless, it was stronger inclined than in *Moschus*. In respect to the processus coronoideus the processus condylaris reaches further laterally than in *C*. *elaphus* and in *Hispanomeryx* [15], as is also the case in *Moschus*. Fossa pterygoidea and foramen mandibulae are not preserved in any of the specimen.

**Fig 12:**
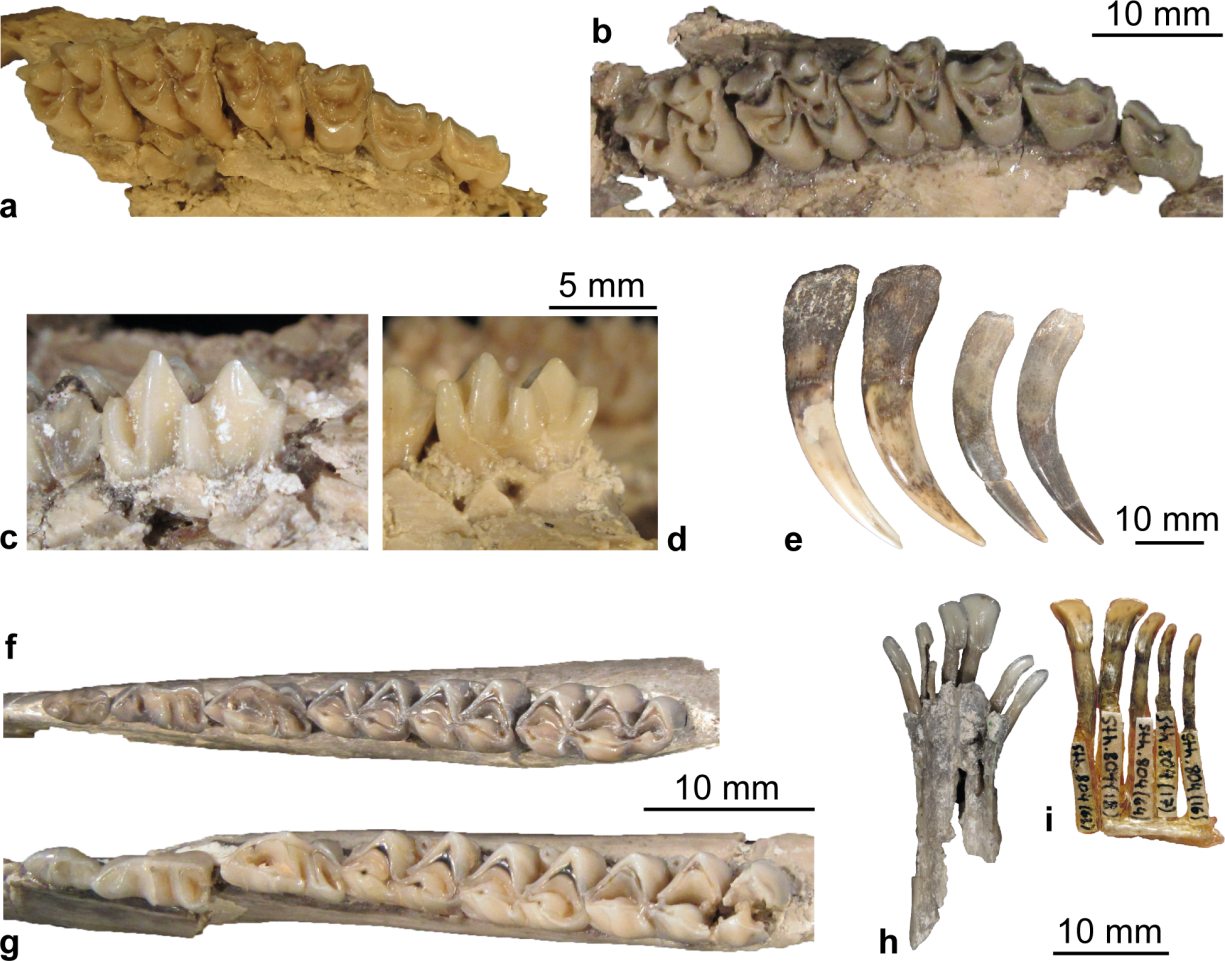
Dentition of Moschidae from Steinheim a. **A.** (a) P2-M3 dex. of NMB Sth. 834, *M*. *flourensianus* from Steinheim a. A., in occlusal view; (b) P2-M3 dex. of SMNS 40010, *M*.? *eiselei*, in occlusal view; (c) labial view of M3 dex of SMNS 40010, *M*.? *eiselei*; (d) labial view of M3 dex of NMB Sth.834, *M*. *flourensianus* from Steinheim a. A.; (e) from left to right: C dex. in lingual view of NMB Sth. 833, *M*.? *eiselei*; C sin. in labial view of NMB Sth. 833, *M*.? *eiselei*; C dex. in lingual view of *M*. *flourensianus* from Steinheim a. A., SMNS 46123; C sin. in labial view of *M*. *flourensianus* from Steinheim a. A., SMNS 46123; (f) p2-m3 dex. of SMNS 46123, *M*. *flourensianus* from Steinheim a. A., in occlusal view; (g) p2-m3 dex. of SMNS 40010, *M*.? *eiselei*, in occlusal view; (h) i1-c sin. and i3-c dex. of SMNS 40010, *M*.? *eiselei*, in linguocclusal view; (i) i1 sin. and i1-c dex. of NMB Sth.804, *M*. *flourensianus* from Steinheim a. A., in linguocclusal view.

Dentition (Figs 3, 11 and 12). *Micromeryx flourensianus* from Steinheim a. A. shows a typical moschid dentition with a closed or nearly closed anterior valley in the triangular lower p4 and a bicuspid third lobe with a high entoconulid in the lower m3. The tooth formula is 0133/3133. In size the teeth are in the upper range of *M*. *flourensianus* from Sansan, or slightly larger, but smaller than *M*.? *eiselei*.

The upper canines are elongated and saber like in males (Fig 12E), and smaller and teardrop shaped in females. The upper canines of the males are smaller than in *M*.? *eiselei* and clearly shorter than in the modern *Moschus*. The anterior upper premolars are elongated triangular with two lingual cones and relatively pronounced labial styles and ribs (Fig 12A). The P3 is often shorter and generally wider than the P2. In comparison to *M*. *flourensianus* from Sansan there is a tendency in the reduction of the lingual crown elements. The P4 is shorter and wider than the anterior premolars and possesses a relatively flat labial wall with moderately pronounced elements comparable to the condition in Sansan. In most of the specimens in Steinheim a. A. a split in anterolingual and posterolingual cone is indicated by a depression on the lingual wall and some specimens show a complex morphology of the fossa with a clear central fold and further additional crests. In *M*. *flourensianus* from Sansan a very weak depression is only present in some specimens and the morphology of the fossa is usually less complex. The upper premolars in *M*. *flourensianus* from Steinheim a. A. are more elongated than in *Moschus* and *Hispanomeryx* [15]. The upper molars (Fig 12A and 12C) are selenodont, trapezoid to subquadratic in shape and possess four main cusps. The morphology is comparable to *M*. *flourensianus* from Sansan. A tendency to increased complexity can be observed with the occurrence of additional crests in many specimens, however. The lingual walls in *M*. *flourensianus* from Steinheim a. A. are stronger inclined than in *Hispanomeryx* and the ribs and styles are not column like, as it is usually the case in the latter [15]. The rib at the metacone and the metastyle are weak.

There is a disparity in the incisor arcade with a clearly widened i1, a slightly widened i2 and less widened i3 and c (visible in the isolated i-c of NMB Sth.804 (Fig 12I)) in contrast to the modern *Moschus*, where the incisor arcade is more homogenous. The lower canine is incisiform in *M*. *flourensianus* from Steinheim a. A.

The lower premolar tooth row is less shortened in *M*. *flourensianus* from Steinheim a. A. than in *Moschus* and *Hispanomeryx* [15]. In comparison to *M*. *flourensianus* from the type locality Sansan the premolars often appear more bulky and shorter; the transitions between the populations are indistinct, however. The p3 is more elongated with a less developed mesolingual conid in *M*. *flourensianus* from Steinheim a. A. than in *Moschus*. As mentioned above the anterior valley in the p4 is closed, or nearly closed in all specimens of *M*. *flourensianus* from Steinheim a. A. as in all Moschidae. The p4s are similar in morphology to the material from the type locality Sansan. In some specimens from Steinheim a. A., the posterior cristid is more oblique and fuses with the transverse cristid, which is not the case in any of the specimens from Sansan. It could represent an advanced stage of molarization of the p4 and slightly reminds of the condition in the p4 of *Moschus*. The p4s of *M*. *flourensianus* from Steinheim a. A. differ from *Hispanomeryx* and *Moschus* in a more elongated shape and a weaker posterior valley, as well as a weaker posterolabial groove (data from [15] and personal observation). Furthermore, the posterolingual cristid is more complex in *Moschus*. *M*. *styriacus* Thenius, 1950 is larger and the p4 is more elongated and shows a stronger posterolabial groove. *M*.*flourensianus* from Steinheim a. A. has mesodont lower molars with a clearly developed external postprotocristid (in form of an additional crest), distinguishing it from *M*. *azanzae*, *M*. *soriae* Sánchez, Domingo and Morales, 2009, *M*. *mirus* Vislobokova, 2007, *Hispanomeryx*, and *Moschus*, where the external postprotocristid is either not present or has a different shape (data from [15,19,20] and personal observation). The tooth crown height, best observable in the lower molars, is slightly increased in *M*. *flourensianus* from Steinheim a. A. in comparison to the material from Sansan; however, the differences are gradual in this case as well and there is no distinct difference between the two assemblages. The lingual wall of the lower molars in *M*. *flourensianus* from Steinheim a. A. is less aligned and more bulky than in *Hispanomeryx*. Furthermore, the lingual elements are more pronounced than in the latter [15]. The anterior cingulid is weaker and lower than in *Moschus* and not fused with the mesostylid as in the latter. For more details on the differences in the dentition to *M*.? *eiselei* see below in description of this species.

### *Micromeryx*? *eiselei* sp. nov.

urn:lsid:zoobank.org:act:28C0018F-32AF-4345-9312-AF525744A735

v 2002 *Hispanomeryx* sp. Heizmann and Reiff

pars 2014 *Micromeryx flourensianus* Costeur

2011 *Micromeryx flourensianus* Costeur

Derivatio nominis: The species is named after Dieter Eisele, former major (1972–2002) of the community of Steinheim am Albuch, for his continuous interest in palaeontological and geological research in the Steinheim basin, and especially for his support of the excavations in the community sandpit from 1969–1982, as well as for establishing the local Meteorkrater-Museum.

**Fig 13:**
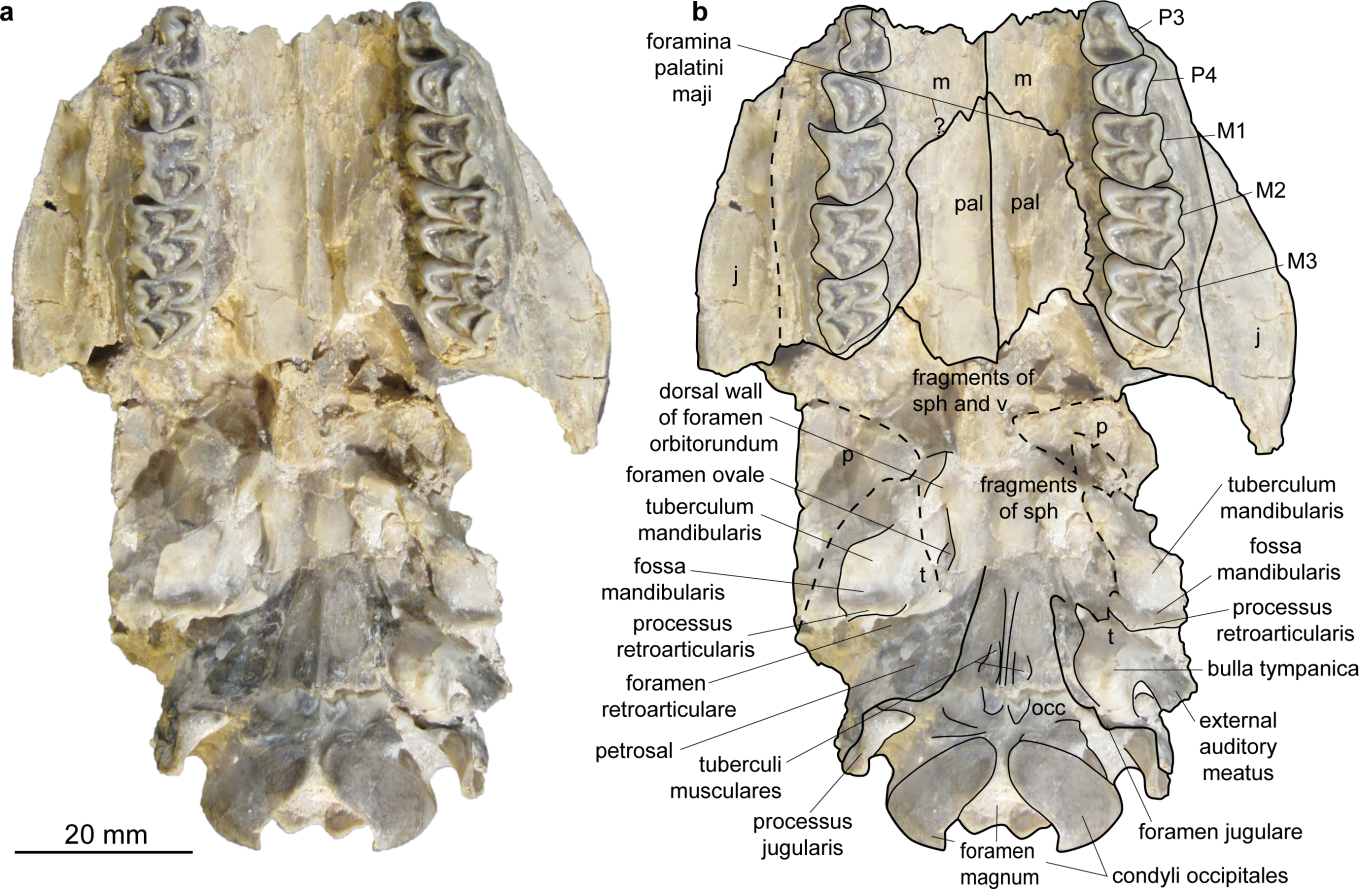
Skull of *Micromeryx*? *eiselei* from Steinheim a. **A.** (a) ventral view of NMB Sth. 833; (b) same as a with labelling of anatomic features; see material and methods for abbreviations.

**Fig 14:**
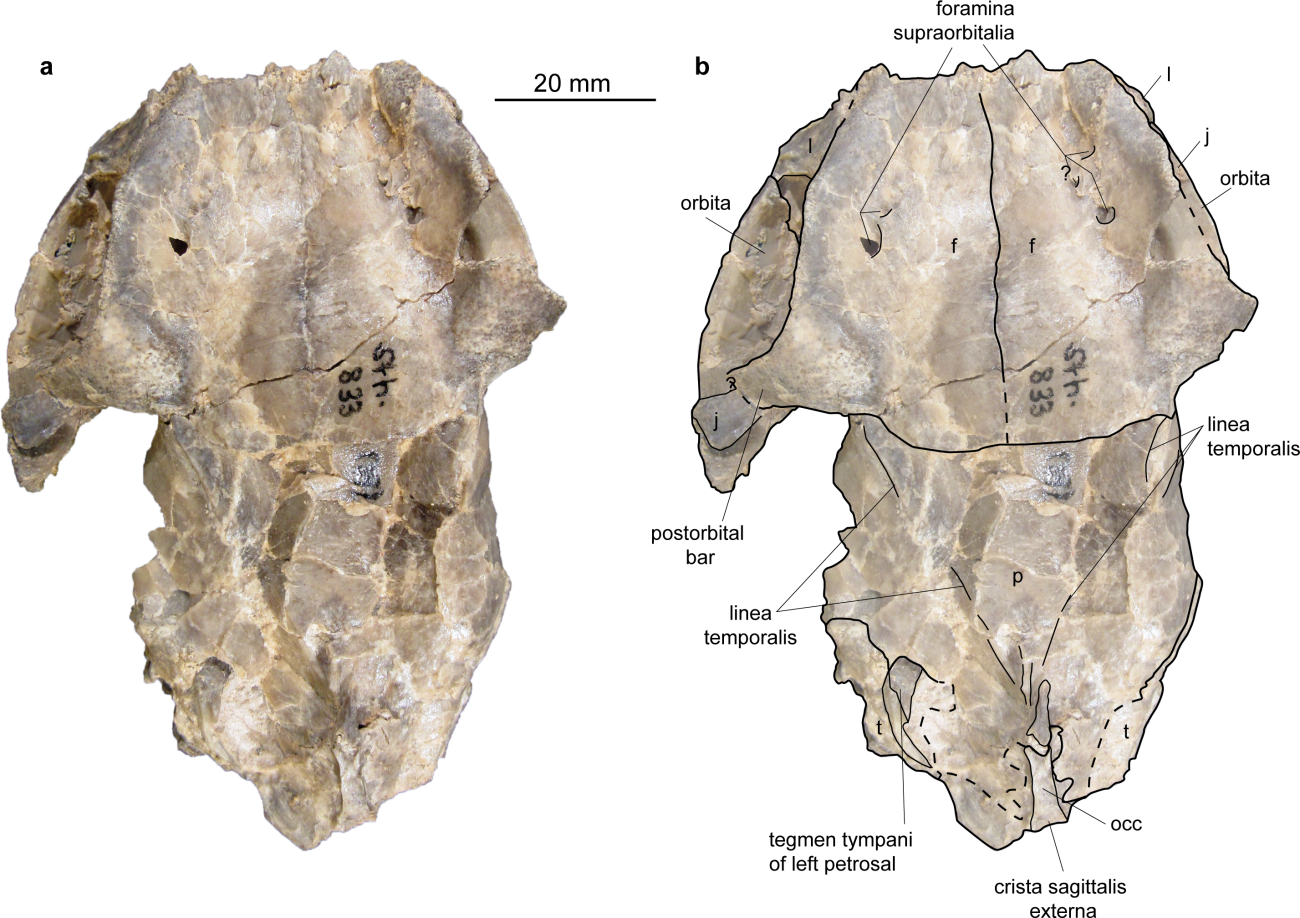
Skull of *Micromeryx*? *eiselei* from Steinheim a. **A.** (a) dorsal view of NMB Sth. 833; (b) same as a with labelling of anatomic features; see material and methods for abbreviations.

**Fig 15:**
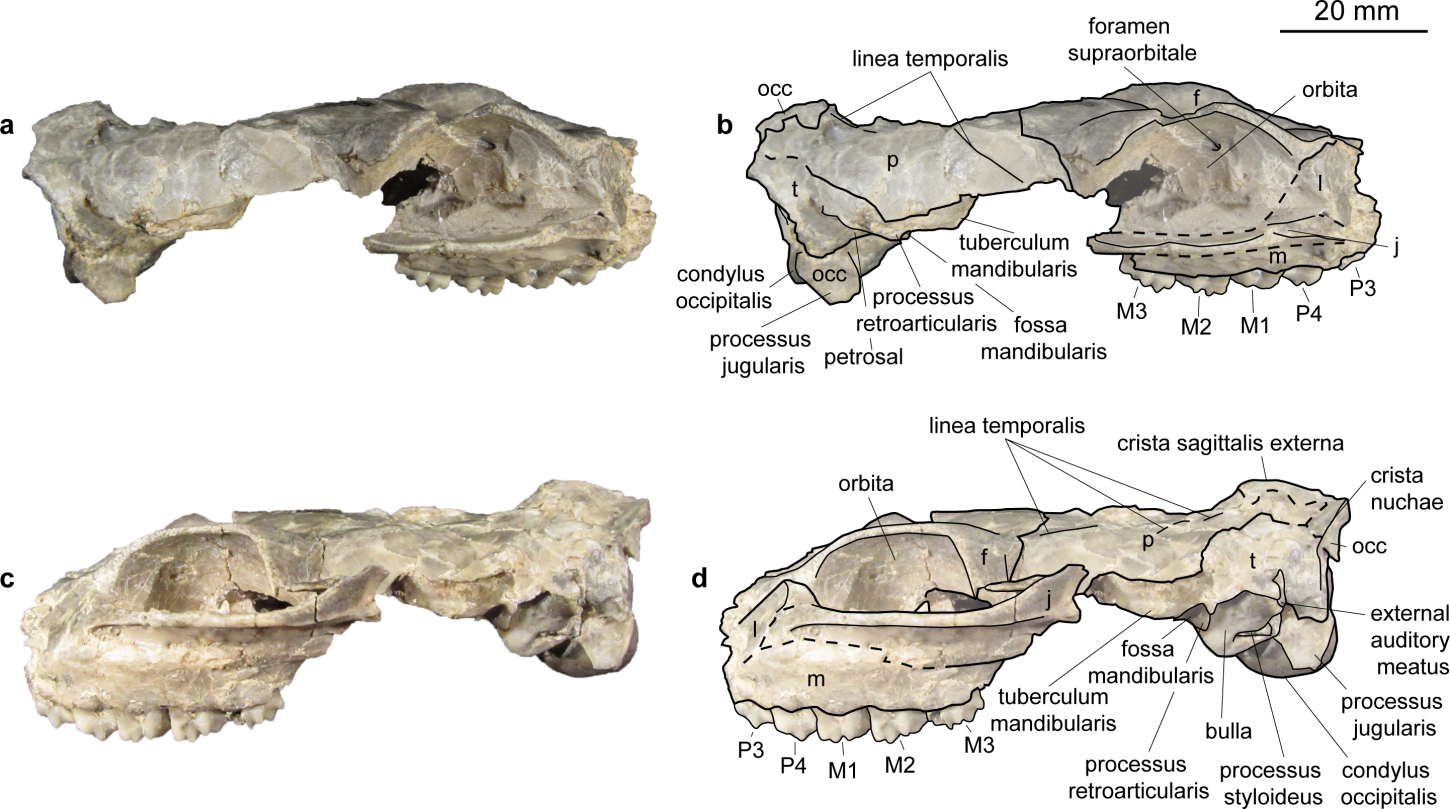
Skull of *Micromeryx*? *eiselei* from Steinheim a. **A.** (a) dextral view of NMB Sth. 833; (b) same as a with labelling of anatomic features; (c) sinistral view of NMB Sth. 833; (d) same as c with labelling of anatomic features; see material and methods for abbreviations.

**Fig 16:**
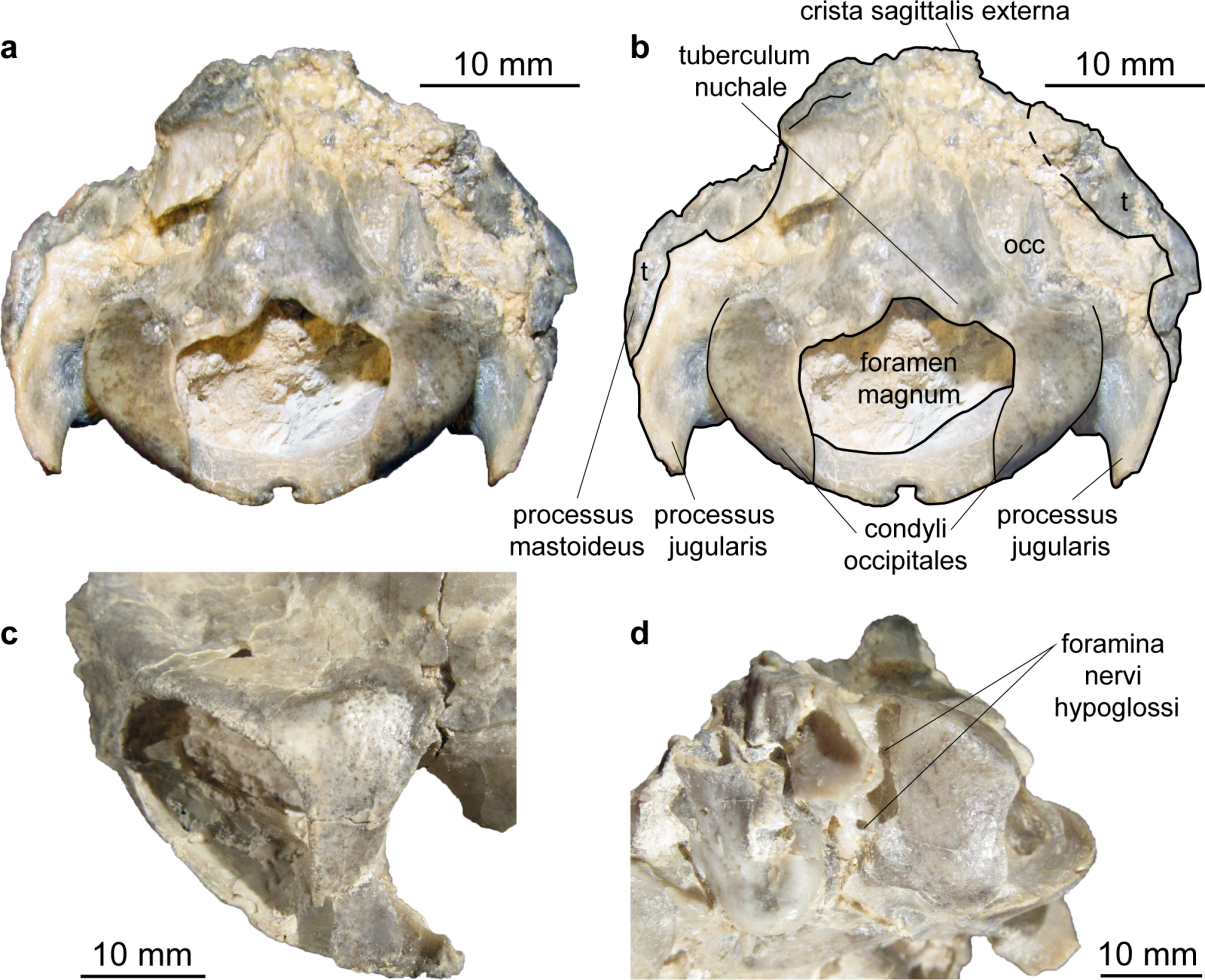
Skull of *Micromeryx*? *eiselei* from Steinheim a. **A.** (a) occipital view of NMB Sth. 833; (b) same as a with labelling of anatomic features; (c) focus on postorbital bar of NMB Sth. 833; (d) focus on foramina for the nervus hypoglossus of NMB Sth. 833; see material and methods for abbreviations.

Holotype: NMB Sth. 833 (partial skeleton with fragmented and compressed skull (described here; Figs 13–16); 3D model of skull, petrosal and inner ear available from [47]).

Paratypes: SMNS 40010 (partial skeleton), SMNS 40617 (P4-M3 sin.), NMB Sth. 825 (fragmented upper molar tooth row), and NMB Sth.162a (m1-2 dex.)

#### Material considered in this analysis

NMB Sth.833 (skull and dentition (inner ear data were considered in [30]), and SMNS 40010 (skull and dentition), SMNS 40617, NMB Sth.825, and NMB Sth.162a. NMB Sth.833 (skull dorsoventrally compressed) is an old male with clearly worn teeth and 40010 (skull highly compressed and fragmented) is a young adult female with permanent, but little worn dentition.

#### Diagnosis

With a closed anterior valley in the triangular lower p4 and a bicuspid third lobe with a high entoconulid in the lower m3 *Micromeryx*? *eiselei* shows a typical moschid dentition. The species can be defined by a unique combination of morphometrical characters: It has a large size, possesses a well pronounced external postprotocristid, a distinct lateral extent of the processus coronoideus, and a relatively wide lower p4. It differs from *M*. *flourensianus* from Sansan and from Steinheim a. A. in a larger size, generally slightly higher tooth crowns, a slightly shorter premolar row in relation to the molar tooth row, a stronger relief in the labial wall of the upper P4, a more elongated posterior part of the upper P3, and more pillar-like elements on the labial wall of the upper molars. From *M*. *flourensianus* from Steinheim a. A. it furthermore differs in a crest on the pars basilaris of the occipital, a more slender and more elongated processus jugularis, a less rounded tympanic bulla, as well as in the morphology of the petrosal and of the inner ear. *M*.? *eiselei* clearly differs from *M*. *azanzae*, *M*. *soriae* and *M*. *mirus* in size and the presence of a strong external postprotocristid. From *M*. *styriacus* the species differs by the relatively wider lower p4. From species of the genus *Hispanomeryx* it differs in the lateral extent of the processus coronoideus, a less shortened premolar row, the presence of an external postprotocristid, and the more bulky labial wall of the lower molars as well as in the shape of p4. *M*.? *eiselei* differs from *Moschus* in the arrangement of the foramina for the nervus hypoglossus, the morphology of bulla tympanica, the morphology of the petrosal, the course of the suture line between maxilla and palatine, the position of the foramina mentalia, a less shortened premolar row, the morphology of the p4, the presence of an external postprotocristid in the lower molars, a lower anterior cingulid, and the less increased tooth crown height.

#### Description and comparison

*Micromeryx*? *eiselei* possessed an elongated and low skull shape, with a broadened cranium and a facial part distinctly narrowing to anterior similar to *Micromeryx flourensianus* from Steinheim a. A. and the modern genus *Moschus*. It was only slightly smaller than the modern *Moschus moschiferus*.

**Fig 17:**
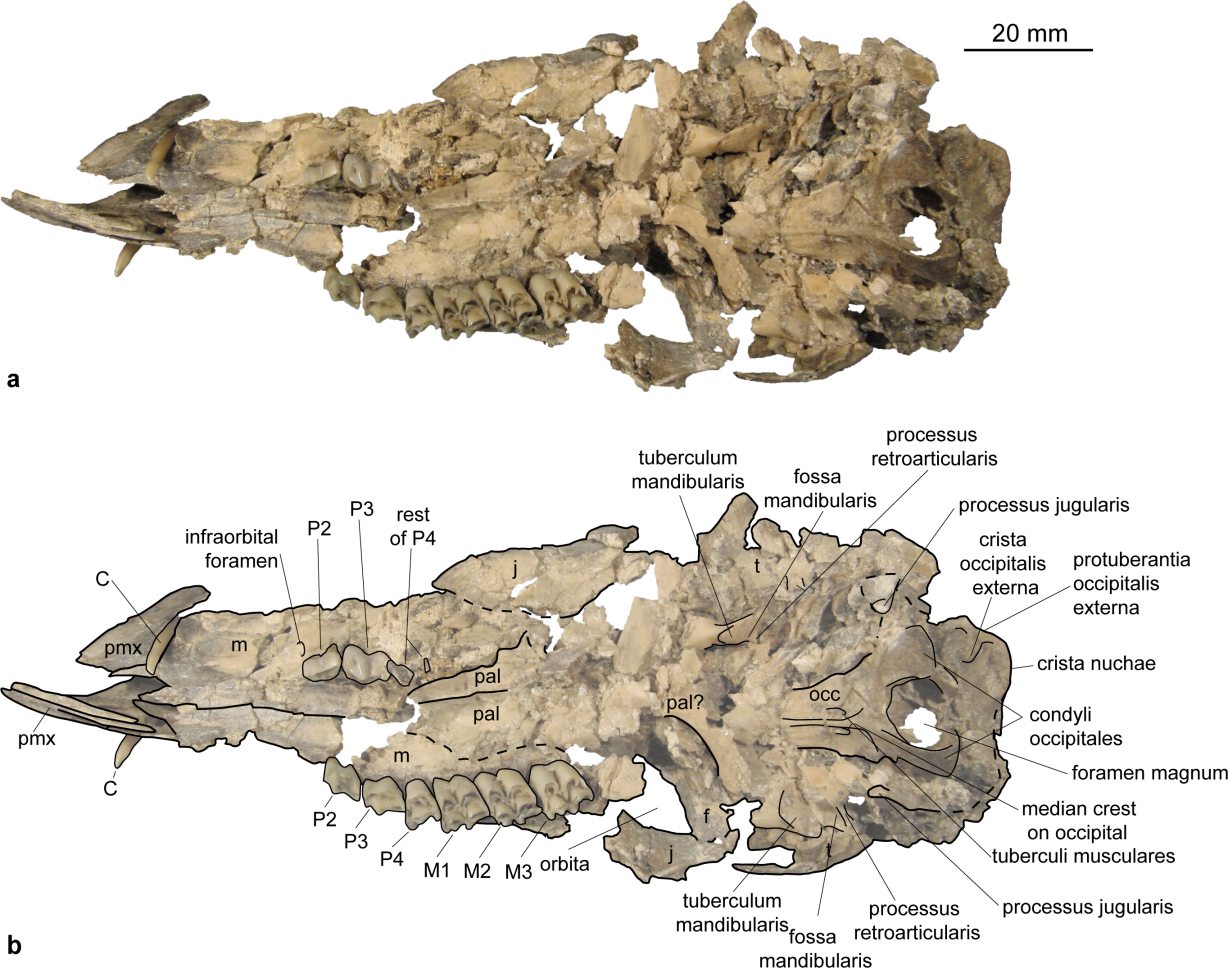
Skull of *Micromeryx*? *eiselei* from Steinheim a. **A.** (a) ventral view of SMNS 40010; (b) same as a with labelling of anatomic features; see material and methods for abbreviations.

**Fig 18:**
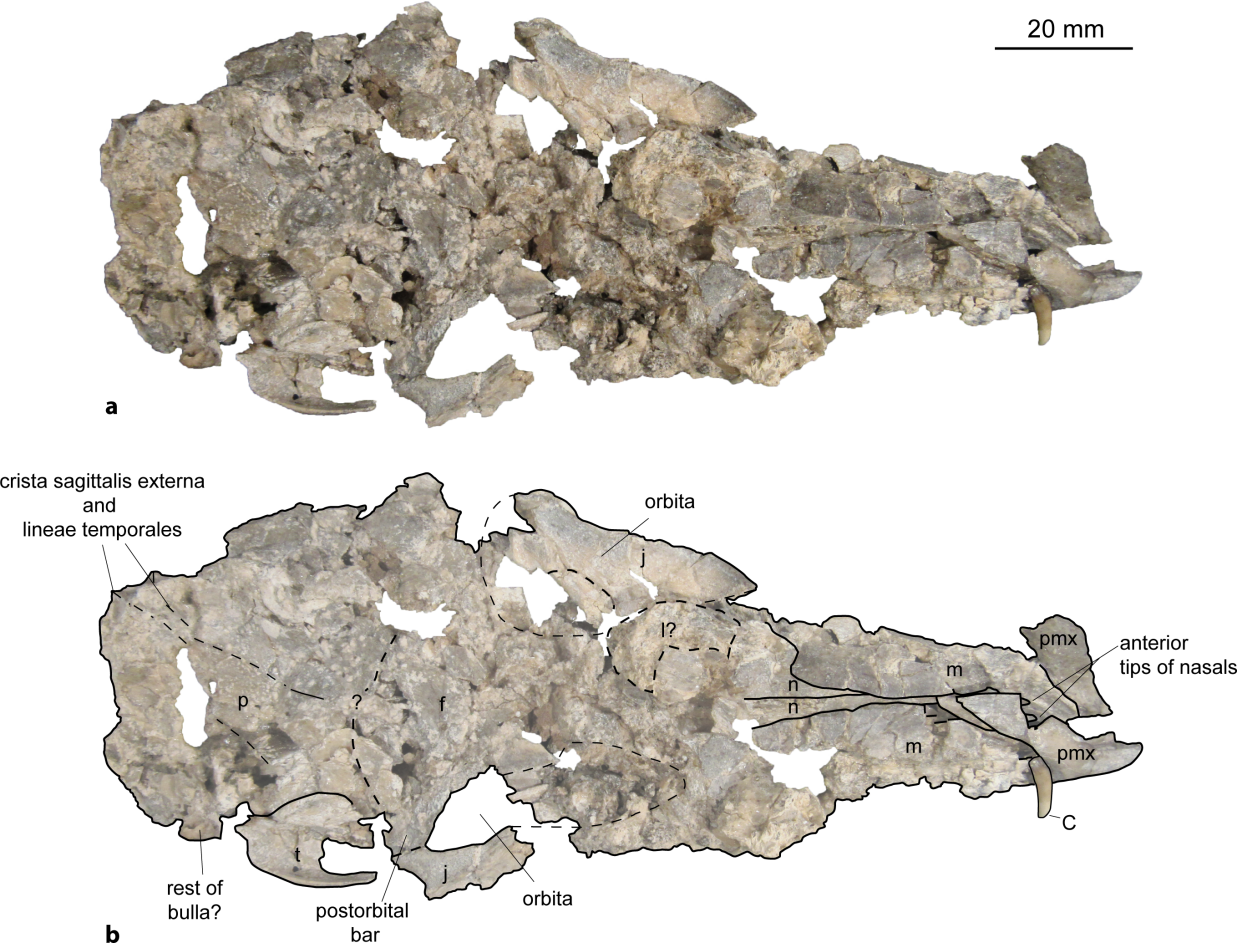
Skull of *Micromeryx*? *eiselei* from Steinheim a. **A.** (a) dorsal view of SMNS 40010; (b) same as a with labelling of anatomic features; see material and methods for abbreviations.

Occipital (Figs 13–18). Parts of the squama occipitalis and of the partes laterales are preserved in NMB Sth.833. A relatively well marked crista sagittalis externa meets the protuberantia occipitalis externa, which is fragmented in NMB Sth.833. As in M. *flourensianus* from Steinheim a. A. a narrow, but distinct crista nuchae proceeds from this point ventrolaterally. Although it is difficult to verify due to fragmentation, the dorsal surface of the condyli occipitales appears slightly more inclined anterodorsally-posteroventrally than in *Moschus*. As in *Moschus* and *Hispanomeryx daamsi* [15] the ventral plane of the condyli is extended more ventrally than the pars basilaris of the occipital. A clearly distinct tuberculum nuchale is positioned on each side of the dorsal rim of the rhomboid shaped foramen magnum in NMB Sth.833. The small foramen jugulare is situated posteromedial of the bulla and not covered by it. As in *M*. *flourensianus* from Steinheim a. A. two foramina are observed in the fossa condylaris ventralis (the latter is quite deep in *M*.? *eiselei;* Fig 16D). And as in *M*. *flourensianus* from Steinheim a. A., the posterior foramen for the nervus hypoglossus is the larger one and is positioned more posterior than in *Moschus*. The processus jugularis is slightly fragmented, but appears to be more slender and elongated than in *M*. *flourensianus* from Steinheim a. A. and stronger developed than in *Moschus*. The surfaces for the muscle insertion are more posterior than it is usually the case in *C*. *elaphus*. The posteromedial tuberculi musculares and the posterior protuberances at the suture to the partes laterales are stronger developed than in *M*. *flourensianus* from Steinheim a. A. (clearly observable in NMB Sth.833). We could not reconstruct the suture line with the basisphenoid. A well pronounced median crest runs along the midline of the pars basalis in *M*.? *eiselei* as in adult *Moschus* specimens (juveniles with deciduous dentition (NMB Sth.5111 and 5731) do not show this crest). As this can be observed in both specimens of *M*.? *eiselei* (SMNS 40010: young female; NMB Sth.833: old male) the feature can neither be ascribed to sexual dimorphism nor to ontogenetic change but appears to be diagnostic for *M*.? *eiselei* in comparison to *M*. *flourensianus* from Steinheim a. A., in specimens with permanent dentition.

Sphenoid (Fig 13). This part of the skull is heavily fragmented in all specimens of *M*.? *eiselei* and no detailed diagnosis can be given on the morphology. The position of the foramen orbitorundum and ovale seem to be roughly similar to the condition in *M*. *flourensianus* from Steinheim a. A.; the presence or absence of a distinct crest in this region cannot be verified, however.

Temporal. Pars squamosa (Figs 13–16). The pars squamosa of the temporal is heavily fragmented and fractured in NMB Sth.833 and SMNS 40010. The course of the suture line to parietal and occipital on the lateral and posterior part of the skull can be reconstructed only arbitrarily; the first ascends from anterolaterally to posteromedially as in *M*. *flourensianus* from Steinheim a. A. and in *Moschus*. Tuberculum articulare and fossa mandibularis (slightly deeper than in *M*. *flourensianus* from Steinheim a. A.) are preserved. Posteriorly a well-developed processus retroarticularis closes the fossa. As in *M*. *flourensianus* from Steinheim a. A. and *Moschus* it terminates in a well pronounced mediolateral crest.

Pars mastoidea and tympanica (Figs 6 and 16). The mastoid process is well pronounced and the temporal forms at least the ventral half of the crista nuchae as in *M*. *flourensianus* from Steinheim a. A., *Moschus* and *Hispanomeryx daamsi* [15]. As in *M*. *flourensianus* from Steinheim a. A. the bulla in NMB Sth.833 is more strongly inflated than in *Moschus*, the ventral surface is rounded, laterally the surface is plainer than in *M*. *flourensianus* from Steinheim a. A. The foramen retroarticulare is covered by the inflated bulla tympanica in NMB Sth.833. As the specimen is compressed, the original situation is difficult to reconstruct, however. In any case, the foramen is quite large, as can be observed on the right side, where the bulla is not preserved in situ. The external auditory meatus is large and subcircular. There is no significant posterior expansion of the bulla, and the tympanohyal vagina is located subposteriorly. The lamina vaginalis does not enclose the processus styloideus and thus the thympanohyal vagina is visible from lateral view. As the lamina is in general quite delicate, we cannot exclude loss due to taphonomic processes, however. The processus styloideus is separated from the processus jugularis of the occipital, comparable to the situation in *Moschus*. As far as it can be reconstructed there is no contact between the tympanic bulla and the occipital. In NMB Sth.833 the anterior wall of the right bulla is preserved as an isolated fragment (Fig 6C). It allows the observation of the inner structure of the bulla. The opening to the external auditory meatus can be observed dorsal of the sulcus tympanicus. Ventrally the hypotympanicum, the tuba eustachii, and the processus muscularis are still preserved. The structure of the hypotympanicum is not cellular.

**Fig 19:**
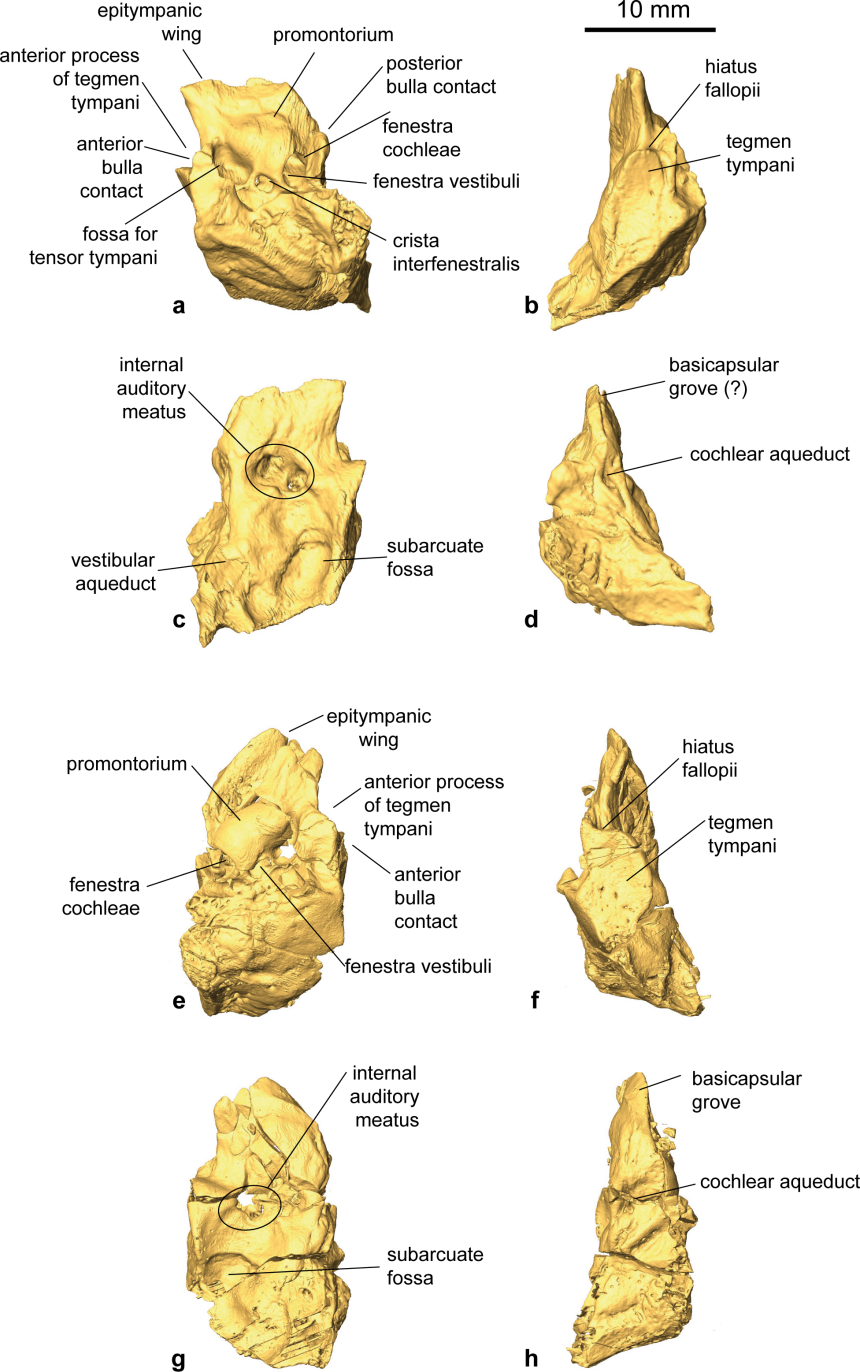
Petrosal of *Micromeryx*? *eiselei* from Steinheim a. **A.** 3D reconstruction of right petrosal of NMB Sth.833 in (a) ventrolateral view; (b) dorsolateral view; (c) dorsomedial view; (d) ventromedial view; 3D reconstruction of left petrosal of SMNS 40010 in (e) ventrolateral view; (f) dorsolateral view; (g) dorsomedial view; (h) ventromedial view.

Pars petrosa and bony labyrinth (Figs 8 and 19). No isolated petrosal is attributed to *M*.? *eiselei*. Its morphology was reconstructed from CT scans of NMB Sth.833 and SMNS 40010. As SMNS 40010 is heavily broken, most of the information comes from NMB Sth. 833. With a general elongated shape and a pointed apex on the epitympanic wing the petrosal is similar in morphology to *M*. *flourensianus* from Steinheim a. A., though slightly less wide in ventromedial-dorsolateral direction. The promontorium has a biconvex hemiellipsoid shape. In contrast to the condition in *M*. *flourensianus* from Steinheim a. A. no weak transpromontorial sulcus can be observed in NMB Sth.833 across the promontorium. Although it is rather unlikely, we cannot fully rule out that the missing of the shallow sulcus could be related to its taphonomical history, as the bone surface appears quite smoothed in the specimen in general. The fenestra cochleae is oval shaped and more than twice as large as the fenestra vestibuli. The crista interfenestralis is of similar width as in *M*. *flourensianus* from Steinheim a. A. The fossa for the tensor tympani is large, deep, elongated (but not as long and narrow as in *Moschus*; see [30]), and bean-shaped, but is not excavated into the tegmen tympani. The posterior articulation to the bulla is larger than in *M*. *flourensianus* from Steinheim a. A. The anterior articulation with the bulla is of similar size as in the latter but larger than in *Moschus*. The ventrolateral plane posterior to it is large and triangular as in *M*. *flourensianus* from Steinheim a. A., but different to e.g. the condition in the cervid *Procervulus*, where it is rectangular [27] and in *Moschus*, where it is elongated and smaller [30]. The anterior process of the tegmen tympani is more delicate than in *M*. *flourensianus* from Steinheim a. A. The tegmen tympani is of similar shape as in *M*. *flourensianus* from Steinheim a. A., but dorsomedial-ventrolateral wider. It shows traces of vascular grooves, as in *Moschus* and *M*. *flourensianus* from Steinheim a. A. A medial protrusion of the pars cochlearis is present. The anteromedial position of the hiatus falloppi is comparable to the condition in *M*. *flourensianus* from Steinheim a. A. There is no knob anterior to the deep subarcuate fossa. Due to the different orientation (see description of inner ear) the vestibular aqueduct is positioned more posterior than in *M*. *flourensianus* from Steinheim a. A. The mastoid region is wedge shaped. The basicapsular groove is less distinct than in *M*. *flourensianus* from Steinheim a. A. and not ventrally oriented. It is separated from the cochlear aqueduct by a bony knob.

The bony labyrinth of NMB Sth.833 is described in [30] and ascribed to *M*. *flourensianus*. Better knowledge of variability in this structure acquired since the first description shows that it is clearly different from *M*. *flourensianus* from Steinheim a. A. described above. The main differences are found in the vestibular aqueduct, which is here longer, straighter, and surmounted by a furrow shaped and long endolymphatic sac, a very different situation than the shorter, curved aqueduct and short pouch shaped sac in *M*. *flourensianus* from Steinheim a. A. The cochlear aqueduct is short, massive and slightly more anteriorly positioned than in *M*. *flourensianus* from Steinheim a. A.

Parietal (Figs 13–15 and 18). The parietal is preserved fragmentarily in NMB Sth.833 and SMNS 40010. The lineae temporales are stronger than in *M*. *flourensianus* from Steinheim a. A. In NMB Sth. 833 the lineae temporales converge posteriorly forming a strong crista sagittalis externa, which proceeds on the occipital. The crista is shorter than in and not as distinct as in *Dremotherium* (see discussion and Fig 2 in [52] for details) but comparable to the condition in *Moschus*, when a crista is present in the latter. However, presence, extent and strength of this structure varies in *Moschus* (a clearly distinct sagittal crest was only observed in SMNS 1238). Interestingly Sánchez and Morales [19] describe a well distinct crista sagittalis in a juvenile male of *Micromeryx azanzae*, whereas in *Hispanomeryx daamsi* a crest is only present in terms of the protuberantia occipitalis externa [15].

Frontal (Figs 14, 15 and 18). The paired frontal bones form the interorbital part of the skull roof, as well as the dorsal and most of the medial wall of the orbitae. As in *M*. *flourensianus* from Steinheim a. A., there are no indications for cranial appendages in any specimen. The supraorbital frontal convexity and the depression anterior to it cannot be reconstructed in detail due to compression. As in *M*. *flourensianus* from Steinheim a. A. they appear similar to the condition in *Moschus*, *Micromeryx azanzae* [19], and in *Hispanomeryx daamsi* [15]. The foramen ethmoideum and the part of the skull with the anterior part of the frontal are not preserved in any of the specimen. Therefore, the presence and extent of the fontanella nasolacrimalis (etmoidal vacuity in [15]; antorbital vacuity in [10]) cannot be reconstructed here as in *M*. *flourensianus* from Steinheim a. A.

The dorsal rim of the orbit is more bulgy in *M*.? *eiselei* (observable in NMB Sth.833) than in *M*. *flourensianus* from Steinheim a. A. As NMB Sth.833 is an older individual, it cannot be excluded that this feature results from its advanced age. The postorbital bar (Fig 16C) is clearly wider than in *Moschus* and *M*. *flourensianus* from Steinheim a. A. The depression posterior to the postorbital bar, ventral to the origin of the linea temporalis, is mediolaterally wider than in *Moschus*. There are two foramina supraorbitalia. The anterior one is smaller and forms a short oblique canal leading into the orbita, while the posterior one is larger and directly opens into the orbita. A distinct sulcus supraorbitalis as in *M*. *flourensianus* from Steinheim a. A. cannot be observed in *M*.? *eiselei*.

Ethmoid. Fragments of the ethmoid can be seen in the CT scans of NMB Sth.833. Specific characteristics of the bone cannot be described.

Nasal (Fig 18). Fragments of the nasal are preserved in SMNS 40010. They are elongated and increase in lateromedial width to anterior. Otherwise, not much can be said on the morphology.

Lacrimal (Figs 14, 15 and 18). The facies facialis of the lacrimal is smooth and slightly concave. It forms the anterior rim of the orbita (preserved in NMB Sth.833). Due to compression and fragmentation the morphology of the facies orbitalis cannot be reconstructed nor can the position and number of foramina lacrimales be verified. However, there is no indication for the presence two foramina on the orbital rim as in cervids.

Jugal (Figs 13–15, 17 and 18). As in *M*. *flourensianus* from Steinheim a. A. the detailed shape and size of the jugal cannot be fully reconstructed due to compression. It forms the ventral rim of the orbit. Like in *Moschus*, *Micromeryx azanzae* [19], and *M*. *flourensianus* from Steinheim a. A. the crista facialis is well marked. It is stronger than in *C*. *elaphus*. It reaches posteriorly as far as the processus temporalis as in *M*. *flourensianus* from Steinheim a. A. The jugal does not form more than 1/3 of the postorbital bar in *M*.? *eiselei* (NMB Sth.834). As only a small part of the processus temporalis of the jugal is preserved, its share on the formation of the temporal bar cannot be reconstructed in detail.

Maxilla (Figs 13–15, 17 and 18). The anterior part of the maxilla is preserved in SMNS 40010 comprising both canines. As SMNS 40010 is a female, the canines are smaller and shorter than in NMB Sth. 833. In ventral view, the anterolateral suture line with the praemaxilla is as well observable in SMNS 40010. Anteriorly the maxillae protrude in between the processi palatini of the praemaxillae and their lateral walls form the posterolateral wall of the fissura palatina. The facies facialis of the maxilla is compressed and fragmented in both specimens. As in *M*. *flourensianus* from Steinheim a. A. it meets the ventral side of the bone anterior of the toothrow forming a sharp and delicate crest (observable in SMNS 40010). The suture line with the jugal can be estimated roughly only in NMB Sth.833. Ventrally of the orbit it descends gradually to posterior. The infraorbital foramen, situated anterior of the anterior root of the P2, is large and oriented anteroposteriorly (observable in SMNS 40010), comparable to the situation in *Moschus*. As in *M*. *flourensianus* from Steinheim a. A. the maxillae meet the palatines at a suture line running from medial at the about the height of the P4 in a curved line posterolaterally. Thus, as in *M*. *flourensianus* from Steinheim a. A., the palatine is stronger extended anteriorly than in *Moschus*. On the left side of NMB Sth.833 the foramen palatinum majus is still preserved on the maxilla at this suture line protruding into the palatine similar to the condition in *M*. *flourensianus* from Steinheim a. A. The maxillary tuberosity is clearly developed.

Praemaxilla (Figs 17 and 18). This element is only preserved in SMNS 40010. In ventrolateral view, it meets the maxilla in a straight line running anteromedial to posterolateral at the height of the canines. It forms the anterior and medial wall of the alveole for the canine and the lateral and medial wall of the fissura palatina. Only fragments of the ventromedial processi palatini are preserved.

Palatine (Figs 13 and 17). Anteriorly bordering the maxilla, the lamina horizontalis of the palatine forms the posterior part of the bony palate including the anterior rim of the choanae. As mentioned above the palatine reaches further anterior than in *Moschus* (see chapter on maxilla for general course of suture line). The suture line with the maxilla meets the concave posterior rim of the bony palate at the height of the M3. In posterior view the fossa pterygopalatina can be observed dorsally of the palate; position and relative size are similar to the condition in *Moschus*.

Vomer (Fig 13). Possible fragments of the vomer are preserved in NMB Sth.833. Statements on shape and posterior extent cannot be given.

Mandibula (Fig 11). A fragmented mandibula is preserved of *M*.? *eiselei* (specimen: SMNS 40010). As in *Moschus*, *C*. *elaphus*, and *M*. *flourensianus* from Steinheim a. A., the corpus mandibulae is slender; the ventral side is slightly less convex than in *M*. *flourensianus* from Steinheim a. A., *Moschus* and *C*. *elaphus*. Only a fragmented pars incisiva is preserved of *M*.? *eiselei*. As in *M*. *flourensianus* from Steinheim a. A. it shows a widening of the pars incisiva to anterior. The length of the symphysis mandibulae and of the margo interalveolaris cannot be estimated. As in *M*. *flourensianus* from Steinheim a. A., a smaller foramen mentale is situated just anterior of the p2, distinguishing this taxon as well from *Moschus*, where this foramen is more anterior, when present. The incisura vasorum facialium seems to have been shallower then in *Moschus*. The angulus mandibulae is fragmented. Shape and extent appear similar as in *Moschus*, however. As in *M*. *flourensianus* from Steinheim a. A. the articulation facet is inclined stronger than in *Moschus*. The processus condylaris reaches further laterally than in *C*. *elaphus* and *Hispanomeryx* [15], as is the case in *M*. *flourensianus* from Steinheim a. A. and *Moschus*. Fossa pterygoidea, foramen mandibulae and processus coronoideus are not preserved in any specimen assigned to *M*.? *eiselei*.

**Fig 20:**
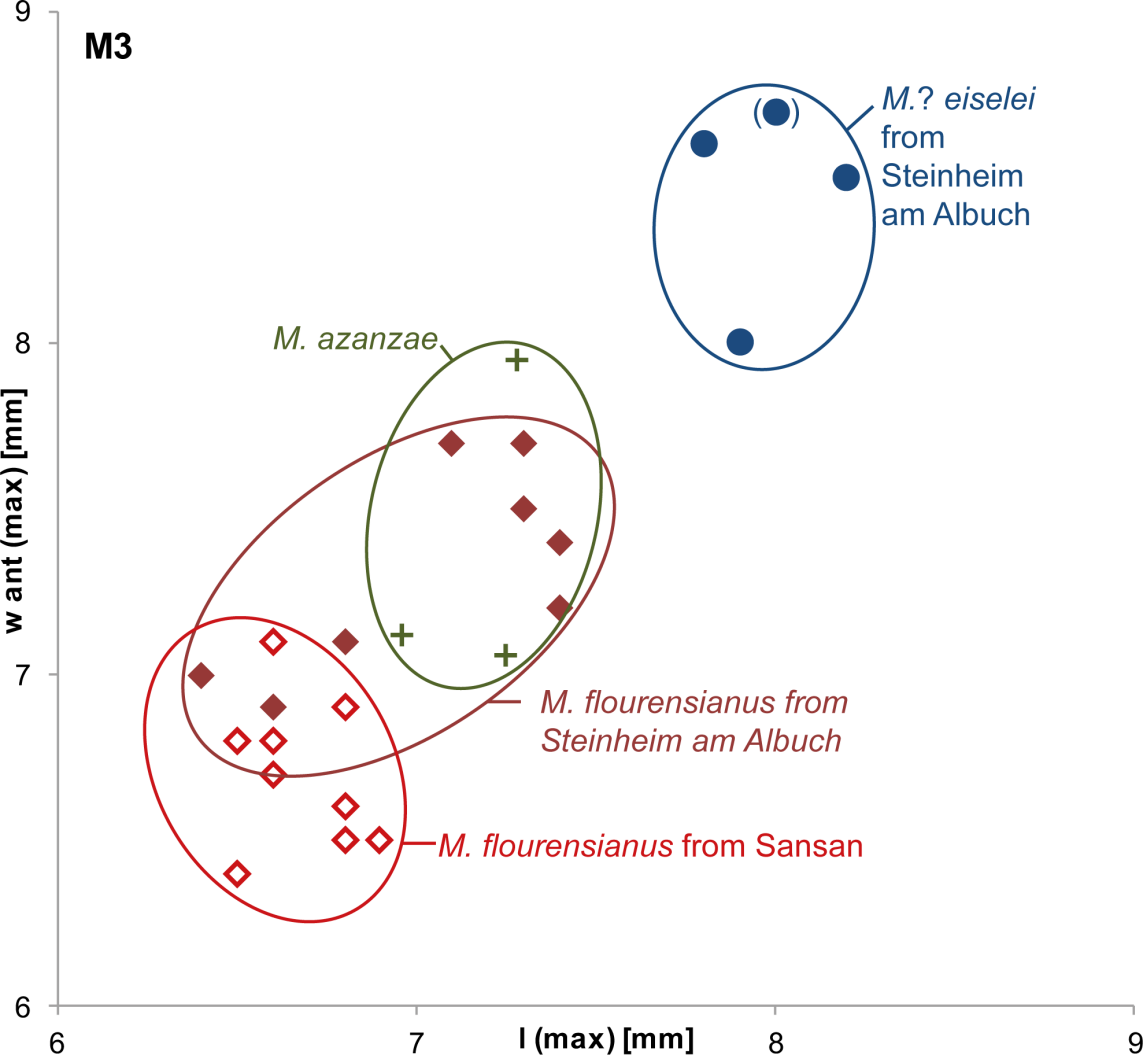
Bivariate plot for dimensions of M3 of Moschidae from Steinheim a. **A.** maximal anterior width (w ant (max)) in mm versus maximal length (l(max)) in mm; light-red non-filled diamonds: *M*. *flourensianus* from the type locality Sansan, dark-red filled diamonds: *M*. *flourensianus* from Steinheim am Albuch, green crosses: *M*. *azanzae* from Spain (after data from [19]), blue circles: *M*.? *eiselei* from Steinheim a. A. (specimen in parentheses posteriorly fragmented; therefore, length measurement is a minimal estimate).

Dentition. *Micromeryx*? *eiselei* possesses a typical moschid dentition with a closed or nearly closed anterior valley in the triangular lower p4 and a bicuspid third lobe with a high entoconulid in the lower m3. The tooth formula is 0133/3133. The teeth of *M*.? *eiselei* are larger than in *M*. *flourensianus* from Steinheim a. A. and from the type locality Sansan (Fig 20). Furthermore, the dentition of the female in *M*.? *eiselei* is larger than the one of the male. This indicates a sexual dimorphism with a larger size in females, as it often occurs in ruminants with a small body size. The strong attrition of the male dentition superposes a clear signal, however. With the description of the postcranial skeleton we hope to gain a better idea about sexual size dimorphism in *M*.? *eiselei*.

As in *M*. *flourensianus* from Steinheim a. A. the upper canines are elongated and saber like in the males (Fig 12E), and smaller and teardrop shaped in the females (Figs 17 and 18). They are shorter than in *Moschus* but longer than in *M*. *flourensianus* from Steinheim a. A.

The anterior upper premolars are elongated triangular with two lingual cones and pronounced labial styles and ribs (Fig 12B). The P3 is often shorter and generally wider than the P2. In the P3 the posterolabial cristid is more elongated in *M*.? *eiselei* than in *M*. *flourensianus* from Steinheim a. A. The P4 is shorter and wider than the anterior premolars, shows only one distinct lingual cone, and due to strong labial styles and a weak rib at the labial cone a concave labial wall. This distinguishes *M*.? *eiselei* from *M*. *flourensianus* from Steinheim a. A., which shows a more flat labial wall and a tendency to split the lingual wall in anterior and posterior cone. The upper premolars are more elongated than in *Moschus* and *Hispanomeryx* [15]. As in *M*. *flourensianus* from Steinheim a. A. the upper molars (Fig 12B and 12D) are selenodont and possess a trapezoid to subquadratic shape with four main cusps. The lingual walls are inclined more strongly than in *Hispanomeryx* and the labial ribs and styles are stronger than it is usually the case in the latter [15]. As in other *Micromeryx* taxa the enamel on the labial wall of the lingual elements is well pronounced, while it is much weaker in the genus *Hispanomeryx*. In contrast to *M*. *flourensianus* from Steinheim a. A. the styles at the labial wall of the upper molars are more pillar-like in *M*.? *eiselei* and the rib at the metacone as well as the metastyle are stronger.

In the fragment of the symphysis of SMNS 40010 it can be observed that the i1 is clearly widened, i2, i3, and c are less wide (Fig 12H). This distinguishes this taxon from the modern *Moschus*, where the incisor arcade is more homogenous, as mentioned above. The lower canine is incisiform in *M*.? *eiselei*, as it is in *M*. *flourensianus* from Steinheim a. A.

Although the premolar tooth row is slightly more shortened in *M*.? *eiselei* than in *M*. *flourensianus* from Steinheim a. A., it is not as shortened as in *Hispanomeryx* [15] and in *Moschus*. The p3 is longer, and possesses a weaker labial incision than it is the case in *Moschus*. It is more slender with a more open anterior valley and a less bulky mesolingual conid than in *M*. *flourensianus* from Steinheim a. A. As typical for moschids the anterior valley in the p4 of *M*.? *eiselei* is closed. It differs from *Hispanomeryx* and *Moschus* in a more elongated shape and weaker posterolabial and -lingual incisions (data from [15] and personal observation). Furthermore, the posterolingual cristid is more complex in *Moschus*. The p4 is wider and shows less distinct posterior incisions than in *M*. *styriacus*. As in *M*. *flourensianus* from Steinheim a. A. *M*.? *eiselei* has mesodont lower molars with a clearly developed external postprotocristid (in form of an additional crest), distinguishing it from *M*. *azanzae*, *M*. *soriae*, *M*. *mirus*, *Hispanomeryx*, and *Moschus*, where the external postprotocristid is either not present or has a different shape (data from [15,19,20] and personal observation). The lingual walls are less flattened and less aligned than in *Hispanomeryx*. The metastylid is slightly stronger than in *M*. *flourensianus* from Steinheim a. A. and in comparison to the latter the anterior cingulid is stronger on the lingual wall. As in *M*. *flourensianus* from Steinheim a. A. the anterior cingulid is weaker and lower than in *Moschus* and not fused with the mesostylid, however.

### Phylogenetic analysis

**Fig 21:**
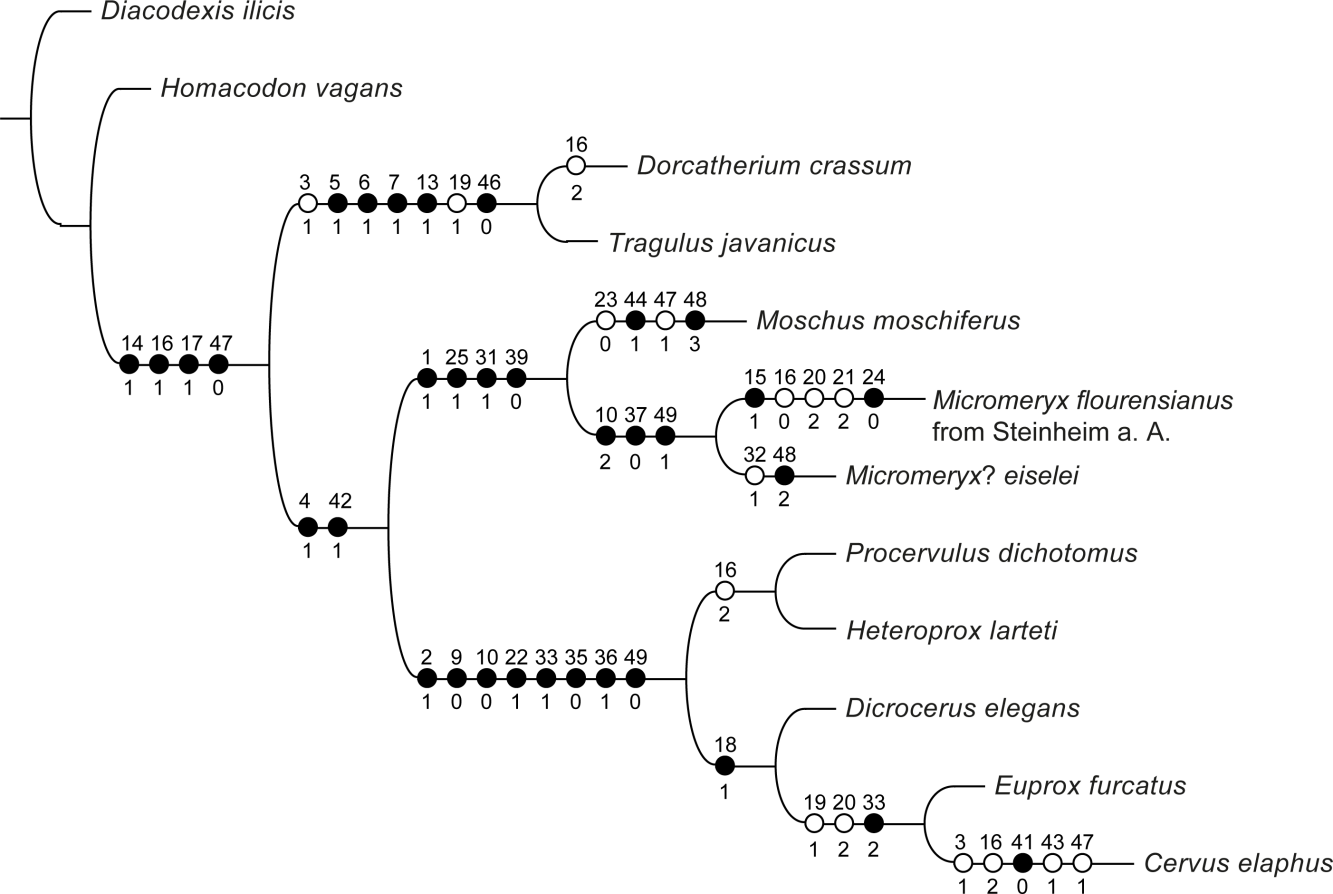
Phylogenetic tree of selected ruminants. The tree is based on 50 characters, comprising 10 characters from the petrosal, 11 from the bony labyrinth, 19 from cranial bones, and 10 from the dentition. A list of unambiguous synapomorphies is given for each node (black circle: strict synapomorphy; white circle: homoplastic synapomorphy). For description of characters, characters states and coding see supporting information.

In order to test the phylogenetic relationships of the two taxa from Steinheim a. A. with the extant moschid *Moschus*, we performed a preliminary phylogenetic analysis with a focus on these taxa (Fig 21). This resulted in a single most parsimonious tree (CI = 0.79; RI = 0.8; for each node, the list of unambiguous synapomorphies is given in Fig 21). Comparable to the results in [27], the monophyly of the clade Ruminantia comprising the tragulids as the sistergroup of the Pecora was confirmed.The Pecora are represented by a cervid clade and a “hornless ruminant clade”, composed by *Moschus* and the taxa form Steinheim a. A. This “hornless ruminant clade” could represent the moschid family. The two taxa from Steinheim a. A. and *Moschus* share the following synapomorphies: an elongated and bean-shaped fossa for the tensor tympani muscle in the petrosal; a less developed dorsal extent of the palatine and a higher sphenoid; a lower parieto-temporal suture; a stronger inclined articulation facet of the mandibula. The two taxa from Steinheim a. A. are monophyletic, being the sister-group of the modern genus *Moschus*, and defined by the following characters: anterior process of the tegmen tympani broad and triangular; maxilla-palatine-suture line on bony palate reaching as anterior as or more anterior than P4; a weaker metastylid.

## Discussion on the taxonomical attribution of the Steinheim a. A. Moschidae

The comparison of the moschids from Steinheim a. A. with other Miocene Moschidae is based primarily on dental material, as comparative descriptions of cranial elements and data from the ear region are limited. Two moschid genera, *Micromeryx* and *Hispanomeryx*, are recorded from the Miocene of Europe and five *Micromeryx*-species are considered valid for this region at the moment: *M*. *flourensianus*, *M*. *styriacus*, *M*. *azanzae*, *M*. *soriae*, and *M*. *mirus*.

### *M*. *flourensianus* from Steinheim a. A.

Taking into consideration data from the literature [3,19,20,46,53–55] and personal observation, *M*. *flourensianus* from Steinheim a. A. differs from *M*. *flourensianus* from the type locality Sansan by an increased tooth size, shorter and more bulky premolars, a more complex lower p4 and upper P4, a tendency towards a more complex pattern in the upper molars, as well as a tendency to increase tooth crown height. However, there is size overlap between the two localities, and the morphology of the dentition is variable within the population from Steinheim a. A. independent of tooth size. With the pending revision of the type material (in preparation) and based on the gradual and indistinct nature of the observed differences in the dentition between *M*. *flourensianus* from Sansan and from Steinheim a. A. we currently cannot justify a separation of the material from Steinheim a. A. from the species *M*. *flourensianus* from Sansan. We therefore assign the specimens from Steinheim a. A. to an "evolved" morphotype of the type species *M*. *flourensianus* for the moment. There are no cranial elements recorded for the type locality.

In any case, *M*. *flourensianus* from Steinheim a. A. differs by size and morphology from the other *Micromeryx*-species. *M*. *mirus* is smaller, possesses higher crowned dentition, and shows a strongly reduced to non-existent external postprotocristid (personal observation and data from [53] and [55]). *M*. *styriacus* is larger (the validity of this taxon is still unclear [19,53]). *M*. *azanzae* does not possess an external postprotocristid [19]. *M*. *soriae* is similar in size to *M*. *flourensianus* from Steinheim a. A. but has a broader external postprotocristid, so far uniquely described for the genus [20]. *M*. *flourensianus* from Steinheim a. A. is smaller than *Hispanomeryx*, and also differs in the presence of an external postprotocristid (not present in any species of the genus *Hispanomeryx*), and the stronger lateral extent of the processus coronoideus [15].

### Defining *Micromeryx*? *eiselei*

In summary the morphologic differences between *M*. *flourensianus* from Steinheim a. A. and *M*.? *eiselei* in the cranium, mandibula, dentition, and especially in the ear region are too large to ascribe them to intraspecific variability. Both taxa are furthermore represented by both sexes and different ontogenetic stages. These differences are sufficient to clearly distinguish between the two species.

The main differences are:

- crest on the pars basilaris of the occipital present in *M*.? *eiselei* but absent in *M*. *flourensianus* from Steinheim a. A.
- processus jugularis more slender in *M*.? *eiselei* than in *M*. *flourensianus* from Steinheim a. A.
- tympanic bulla less rounded in *M*.? *eiselei* than in *M*. *flourensianus* from Steinheim a. A.
- petrosal less wide in ventromedial-dorsolateral direction in *M*.? *eiselei* than in *M*. *flourensianus* from Steinheim a. A.
- posterior bulla articulation in petrosal larger in *M*.? *eiselei* than in *M*. *flourensianus* from Steinheim a. A.
- anterior process of the tegmen tympani more delicate in *M*.? *eiselei* than in *M*. *flourensianus* from Steinheim a. A.
- tegmen tympani dorsomedially-ventrolaterally wider in *M*.? *eiselei* than in *M*. *flourensianus* from Steinheim a. A.
- course of the vestibular aqueduct with respect to the common crus parallel in *M*.? *eiselei*, divergent in *M*. *flourensianus* from Steinheim a. A.
- vestibular aqueduct longer in *M*.? *eiselei* than in *M*. *flourensianus* from Steinheim a. A.
- short pouch shaped endolymphatic sac triangular in *M*. *flourensianus* from Steinheim a. A. in contrast to straight long endolymphatic sac in *M*.? *eiselei*
- general tooth size larger in *M*.? *eiselei* than in *M*. *flourensianus* from Steinheim a. A.
- posterior part of the upper P3 slightly more elongated in *M*.? *eiselei* than in *M*. *flourensianus* from Steinheim a. A.
- labial wall of upper P4 with stronger relief in *M*.? *eiselei* than in *M*. *flourensianus* from Steinheim a. A.
- labial wall of upper molars more pillar-like in *M*.? *eiselei* than in *M*. *flourensianus* from Steinheim a. A.
- p3 slightly more slender with a more open anterior valley and a less bulky mesolingual conid in *M*.? *eiselei* than in *M*. *flourensianus* from Steinheim a. A. (less distinct feature)
- metastylid and anterior cingulids stronger in the lower molars of *M*.? *eiselei* than in the ones of *M*. *flourensianus* from Steinheim a. A. (less distinct feature).

Both taxa differ from the genus *Moschus* in:

- the arrangement of the foramina for the nervus hypoglossus
- the course of the suture line between maxilla and palatine
- the morphology of the petrosal
- the position of the foramina mentalia
- a less shortened premolar row
- a simpler morphology in the p4
- the presence of an external postprotocristid
- less increase in tooth crown height.

There are however characters shared by *M*.? *eiselei* and *Moschus* distinguishing both from *M*. *flourensianus* from Steinheim a. A. These are:

- the crest on pars basilaris of the occipital
- the parallel course of the vestibular aqueduct with respect to the common crus
- the longer vestibular aqueduct
- an elongated endolymphatic sac.

*Micromeryx*? *eiselei* from Steinheim a. A. cannot be assigned to any known *Micromeryx*- or *Hispanomeryx*-species. It possesses a unique combination of characters in cranial, mandibular, and dental morphology: large size of teeth, the presence of a strongly pronounced external postprotocristid, a distinct lateral extent of the processus coronoideus, and a wider p4 than that observed in *M*. *styriacus*. Therefore, we consider it reasonable to erect a new species, although the postcranial material has not been analysed yet. *M*.? *eiselei* clearly differs from *Moschus* in the morphology of the cranium and the dentition. Interestingly, the characters shared by *M*.? *eiselei* and *Moschus* are only characters of the ear region and the basicranium, which are considered to be less plastic than e.g., dental data. However, considering the evolution of Pecora, some characters of the ear region are plesiomorphic [27], and thus non-informative for the relative position of these two species in the phylogenetic tree. These features are still an indication, however, that with more data of different Moschidae from the basicranium and ear region, combined with data on the complete skeleton, the sister group relationship of *M*. *flourensianus* from Steinheim a. A. and *M*.? *eiselei* might prove less stable than it now appears. However, the revision of the type material of *M*. *flourensianus* as well as a full analyses of the postcranial and the ear regions of more fossil Moschidae is in progress, but not yet completed. Therefore, we can assign *M*.? *eiselei* to a new species but not to a new genus, and thus decided with reservations to leave it in the genus *Micromeryx* at present.

## Conclusion

The small ruminant remains from the Middle Miocene locality Steinheim a. A. can be assigned to Miocene Moschidae closely related to the living genus *Moschus*. Dental and cranial characters as well as features of the ear region reveal the presence of at least two species at the locality.

The smaller of the two, *Micromeryx flourensianus* from Steinheim a. A., is similar to the type material of *M*. *flourensianus* from Sansan, but differs by the presence of an “evolved” dentition. The larger taxon, *Micromeryx*? *eiselei* clearly differs from all Miocene Moschidae and is therefore referred to a new species.

The two moschid taxa described here from Steinheim a. A. originate from the same layer in the stratigraphic column based on the biostratigraphy of the embedded gastropods. The fossil assemblage shows a high percentage of semi-articulated partial skeletons and is embedded in lacustrine sediments, it did not result from a fluvial accumulation and represents an autochthonous taphocoenosis. Thus, independent of the further taxonomic status, there are two sympatric moschid species recorded from Steinheim a. A. This confirms once more that the co-occurrence of two moschid species is rather a more common than an exceptional phenomenon for the Miocene. However, it is the first time that this observation is made with unambiguous remains for European material outside of Spain (for the locality Gratkorn (late Middle Miocene, Austria) fragmentary teeth were tentatively assigned to? *Hispanomeryx* due their size and described as sympatric with *Micromeryx flourensianus* by Aiglstorfer et al. [46]; with the here published remains from Steinheim am Albuch we show that there are more large sized moschids in the Miocene of Europe; thus the cautiousness given in the cited publication is fully justified and the teeth could represent a different taxon after all).

The exhaustive description of the morphology of the ear region combined with data on cranial, mandibular, and dental material represented here is the first step towards a better understanding of the possibly oldest moschid species, *M*. *flourensianus* and the Central/Western European record of the family. Combining the data published here with the study of the type material, and postcranial remains, as well as with data for the ear region of more Miocene Moschidae will improve the knowledge on the early evolutionary history of the family and the origin of the recent genus *Moschus*.

## Supporting information

S1 Text Character list of phylogenetic analysis. (DOCX)

S1 File Character coding of phylogenetic analysis. (NEX)

S1 Table Dental measurements for *Micromeryx flourensianus* from Steinheim am Albuch. (XLSX)

S2 Table Dental measurements for *Micromeryx*? *eiselei* from Steinheim am Albuch. (XLSX)

## Abbreviations

GPIT: Paläontologische Sammlung der Universität Tübingen, Tübingen, Germany
MNHN: Muséum national d’histoire naturelle, Paris, France
SMNS: Staatliches Museum für Naturkunde Stuttgart, Stuttgart, Germany
NMB: Naturhistorisches Museum Basel, Basel, Switzerland
UMJGP: Universalmuseum Joanneum, Graz, Austria
sin: left
dex: right
aa: asc ampulla
asc: anterior semicircular canal
ca: cochlear aqueduct
cc: common crus
co: cochlea
es: endolymphatic sac
f: frontal
fc: fenestra cochleae
fv: fenestra vestibuli
j: jugal
l: lacrimal
la: lsc ampulla
lsc: lateral semicircular canal
m: maxilla
n: nasal
occ: occipital
p: parietal
pa: psc ampulla
pal: palatine
pl: primary lamina
pmx: premaxilla
psc: posterior semicircular canal
pt: pterygoid
sac: saccule
sl: secondary lamina
sph: sphenoid
t: temporal
ut: utricule
v: vomer
va: vestibular aqueduct
i: lower incisor
C/c: upper/lower canine
P2/p2: upper/lower second premolar
P3/p3: upper/lower third premolar
P4/p4: upper/lower forth premolar
M1/m1: upper/lower first molar
M2/m2: upper/lower second molar
M3/m3: upper/lower third molar
