# Supplementary material for: Micromeryx? eiselei—A new moschid species from Steinheim am Albuch, Germany, and the first comprehensive description of moschid cranial material from the Miocene of Central Europe
Source: PLoS One. 2017 Oct 16;12(10):e0185679. doi: 10.1371/journal.pone.0185679 (PMC5642927; doi:10.1371/journal.pone.0185679)
Supplement: S1 Text — (DOCX) [file pone.0185679.s001.docx]

Character list

Petrosal Bone

1. Shape of the fossa for the tensor tympani muscle: round to square (0); elongated and bean-shaped (1) (from [[1](#_ENREF_1)]).

2. Extent of the fossa for the tensor tympani muscle: not excavated into the tegmen tympani (0); excavated into the tegmen tympani (1) (from [[2](#_ENREF_2)]).

3. Transpromontorial sulcus: present (0); absent (1) (from [[2](#_ENREF_2)]).

4. Medial protrusion of the pars cochlearis: absent (0); present (1) (from [[2](#_ENREF_2)]).

5. Knob anterior to the subarcuate fossa: absent (0); present (1) (from [[1](#_ENREF_1)]).

6. Subarcuate fossa: shallow (0); deep (1) (from [[2](#_ENREF_2)]).

7. Shape of mastoid region: wedge (0); knob (1) (from [[2](#_ENREF_2)]).

8. Basicapsular groove: absent (0); dorsal (1); on the edge of the epitympanic wing (2) (modified from [[2](#_ENREF_2)]).

9. Anterior process of the tegmen tympani: not protruding (0); protruding (1) (this study).

10. Anterior process of the tegmen tympani: broad and ovoid (0); pointed pilar (1); broad and triangular (2) (this study).

Bony Labyrinth

11. Number of cochlear turns: equal or less than two (0); between two and three (1); equal or more than three (2) (modified from [[3](#_ENREF_3)]).

12. Insertion of the lateral semicircular canal in the vestibule towards the posterior ampulla: low in posterior ampula (0); high dorsally between posterior ampula and commun crus (1); high in posterior ampula (2); anterior to posterior ampula in vestibule (3) (modified from [[3](#_ENREF_3)]).

13. Extension of the lateral semicircular canal with respect to the plane of the posterior semicircular canal in dorsal or lateral view: no extension beyond the plane (0); extension beyond the plane (1) (modified from [[3](#_ENREF_3),[4](#_ENREF_4)]).

14. Secondary common crus: present (0); absent (1) (from [[4](#_ENREF_4)]).

15*.* Course of the vestibular aqueduct with respect to the common crus: parallel (0); diverging (1) (from [[1](#_ENREF_1)]).

16. Length of the vestibular aqueduct: less than the common crus (0); same as common crus (1); longer than common crus (2) (from [[1](#_ENREF_1)]).

17. Size of the endolymphatic sac: small (0); large (1) (from [[1](#_ENREF_1)]).

18. Fusion of the lateral semicircular canal with posterior ampulla: absent (0); partial to complete fusion (1) (from [[5](#_ENREF_5)]).

19. Relative thickness of the basal cochlear turn: thick (0); thin (1) (from [[5](#_ENREF_5)]).

20. Section of the cochlear aqueduct: incipious (0; )flat (1); ovoid to circular (2) (from [[5](#_ENREF_5)]).

21. Shape of the endolymphatic sac: knob-like (0); straight and funnel-like (1); triangular in shape (2); pouch-like (3) (from [[1](#_ENREF_1)]).

Cranium and Mandibula

22) Shape of processus jugularis: short and triangular, not reaching further ventral than condyli occipitales (0), long and slender, reaching further ventral than condyli occipitales (1) (this study).

23) Condition of the foramina of the nervus hypoglossus in fossa condylaris: small foramen, if present, posterior to main foramen and the latter is not shifted to posterior (0), small foramen, if present, anterior to main foramen and the latter is clearly shifted to posterior (1) (this study).

24) Median ridge on ventral surface of basioccipital in adults: absent (0); present (1) (this study).

25) Dorsal extend of the palatine and height of sphenoid: dorsal extend of palatine more developed (clearly above height of ventral rim of orbita in lateral view) and reduced height of the sphenoid (0); dorsal extend of palatine less developed (not above height of ventral rim of orbita in lateral view) and higher sphenoid (1) (this study).

26) Condition of the tympanohyal vagina: reduced enclosing, affecting maximally only to the proximal third part of the tympanohyal (0); complete enclosing, the tympanohyal vagina is not visible in lateral view (1) (from [[6](#_ENREF_6)]; character-list 2).

27) Condition of the tympanic bulla: posteriorly not or only little inflated (0); posteriorly moderately, laterally stronger inflated (1); posteriorly and laterally strongly inflated (2) (modified from[[6](#_ENREF_6)]; character-list 1).

28) Condition of suprameatal fissure: open (0); closed (1) (from [[6](#_ENREF_6)]; character-list 1).

29) Foramen retroarticulare: covered by the bulla/external auditory meatus (0); not covered by the bulla/external auditory meatus (1) (modified from [[6](#_ENREF_6)]; character-list 1).

30) Occipital/mastoid relationship: well developed mastoid bone that forms part of the nuchal crest along the occipital bone (0); small mastoid that does not form part of the nuchal crest (1) (from [[6](#_ENREF_6)]; character-list 1).

31) Location of the parieto-temporal suture: located between the upper and lower borders of the temporal fossa (0); located near the inferior border of the temporal fossa (1) (from [[6](#_ENREF_6)]; character-list 1).

32) Mediolateral width of the postorbital bar: less flattened, mediolateral width dorsally less than 1/3 of orbita length (0), stronger flattened with mediolateral 1/3 of orbita length or more (1) (this study).

33) Cranial appendage in males: absent (0); present, no modern coronet (1); present, modern coronet (2) (this study).

34) Fontanella nasolacrimalis (ethmoidal vacuity): absent (0); small, less than 2/3 of the facies facialis of the lacrimal (1); large, 2/3 of facies facialis of lacrimal or more (2) (modified from [[6](#_ENREF_6)]; character-list 1).

35) Fossa lacrimalis: present (0); absent (1) (modified from [[6](#_ENREF_6)]; character-list 1).

36) Number and position of foramina lacrimalia: one foramen on facies orbitalis (0); two foramina on margin of orbit (1) (from [[6](#_ENREF_6)]; character-list 1).

37) Maxilla-palatine-sutureline on bony palate: reaching as anterior as or more anterior than P4 (0), reaching not more anterior than M1 (1) (this study).

38) Length of margo interalveolaris: line measured from anterior rim of p2 to posterior rim of symphsysis mandibulae 45 % of postcanine tooth row length or less (0), line measured from anterior rim of p2 to posterior rim of symphysis mandibulae more than 45 % of postcanine tooth row length (1) (this study).

39) Articulation facet in mandibula: stronger inclined (0), less inclined (1) (this study).

40) Articulation facet in mandibula: lateral extension: clearly present (0), weak or absent (1) (modified from [[6](#_ENREF_6)]; character-list 1).

Dentition

41) Condition of the upper canines of males: small, pronounced canines normally absent (0), elongated canines, gradually decreasing in width, broad (width at base enamel equal or more than 0,3 of length of canine (line drawn posteriorly form base enamel to tip) (1); elongated canines, gradually decreasing in width, slender (width at base enamel less than 0,3 of length of canine (line drawn posteriorly form base enamel to tip) (2); elongated canines, with more constant anteroposterior width, slender (width at base enamel less than 0,3 of length of canine (line drawn posteriorly form base enamel to tip) (3) (modified from [[6](#_ENREF_6)]; character-list 1).

42) Morphology of the P4: triangular in shape (0), semicircular in shape (1) (modified from [[6](#_ENREF_6)]; character-list 1).

43) Labial elements in upper molars: pyramidal (0), more pillar-shaped (1) (this study).

44 Incisor arcade: i1 widened anteroposteriorly clearly stronger than i2-c1 (0); anteroposterior width of i1 similar as in i2-c1 (1) (this study).

45) Morphology of the p4: without clearly developed lingual elements (0), strongly developed anterolingual cristid that encloses totally or almost totally the anterior valley and the mesolingual conid joins the transverse cristid through the posterolingual cristid (1), anterior valley open and the mesolingual conid or posterolingual cristid fused with the transverse cristid (2) (modified from [[6](#_ENREF_6)]; character-list 1).

46) External postmetacristid: present (0), absent (1) (this study).

47) External postprotocristid: present (0), absent (1) (modified from [[6](#_ENREF_6)]; character-list 1).

48) Development of the anterior cingulid in the lower molars: not developed (0); weak to moderately developed (1); stronger developed, clearly invading lingual side (2); strong, fused with mesostylid (3) (modified from [[6](#_ENREF_6)]; character-list 1 and 2).

49) Development of the metastylid: well developed (0); weak (1); absent (2) (from [[6](#_ENREF_6)]; character-list 1 and 2).

50) Morphology of the third lobe of the m3: distally open or low wall (0); distally closed with high wall (1) (modified from [[6](#_ENREF_6)]; character-list 2).

References

1. Mennecart B, Costeur L (2016) A *Dorcatherium* (Mammalia, Ruminantia, Middle Miocene) petrosal bone and the tragulid ear region. Journal of Vertebrate Paleontology.

2. O’Leary MA (2010) An anatomical and phylogenetic study of the osteology of the petrosal of extant and extinct artiodactylans (Mammalia) and relatives. Bulletin of the American Museum of Natural History 335: 206.

3. Ekdale EG (2013) Comparative Anatomy of the Bony Labyrinth (Inner Ear) of Placental Mammals. PLOS ONE 8: e66624.

4. Macrini TE, Flynn JJ, Ni X, Croft DA, Wyss AR (2013) Comparative study of notoungulate (Placentalia, Mammalia) bony labyrinths and new phylogenetically informative inner ear characters. Journal of Anatomy 223: 442-461.

5. Mennecart B, Rössner GE, Métais G, DeMiguel D, Schulz G, Müller B et al. (2016) The petrosal bone and bony labyrinth of early to middle Miocene European deer (Mammalia, Cervidae) reveal their phylogeny. Journal of Morphology 277: 1329-1338.

6. Sánchez IM, Domingo MS, Morales J (2010) The genus *Hispanomeryx* (Mammalia, Ruminantia, Moschidae) and its bearing on musk deer phylogeny and systematics. Palaeontology 53: 1023-1047.
